# The Usefulness of the C_2_HEST Risk Score in Predicting Clinical Outcomes among Hospitalized Subjects with COVID-19 and Coronary Artery Disease

**DOI:** 10.3390/v14081771

**Published:** 2022-08-14

**Authors:** Piotr Rola, Adrian Doroszko, Małgorzata Trocha, Damian Gajecki, Jakub Gawryś, Tomasz Matys, Katarzyna Giniewicz, Krzysztof Kujawa, Marek Skarupski, Barbara Adamik, Krzysztof Kaliszewski, Katarzyna Kiliś-Pstrusińska, Agnieszka Matera-Witkiewicz, Michał Pomorski, Marcin Protasiewicz, Marcin Madziarski, Marta Madej, Grzegorz Gogolewski, Goutam Chourasia, Dorota Zielińska, Szymon Włodarczak, Maciej Rabczyński, Janusz Sokołowski, Ewa Anita Jankowska, Katarzyna Madziarska

**Affiliations:** 1 Department of Cardiology, Provincial Specialized Hospital, Iwaszkiewicza 5 Street, 59-220 Legnica, Poland; 2 Clinical Department of Internal and Occupational Diseases, Hypertension and Clinical Oncology, Faculty of Medicine, Wroclaw Medical University, Borowska 213, 50-556 Wroclaw, Poland; 3 Department of Pharmacology, Faculty of Medicine,Wroclaw Medical University, Mikulicza-Radeckiego 2 Street, 50-345 Wroclaw, Poland; 4 Statistical Analysis Centre, Wroclaw Medical University, K. Marcinkowski Street 2-6, 50-368 Wroclaw, Poland; 5 Faculty of Pure and Applied Mathematics, Wroclaw University of Science and Technology, Wybrzeże Wyspiańskiego Street 27, 50-370 Wroclaw, Poland; 6 Clinical Department of Anesthesiology and Intensive Therapy, Faculty of Medicine, Wroclaw Medical University, Borowska Street 213, 50-556 Wroclaw, Poland; 7 Clinical Department of General, Minimally Invasive and Endocrine Surgery, Faculty of Medicine, Wroclaw Medical University, Borowska Street 213, 50-556 Wroclaw, Poland; 8 Clinical Department of Paediatric Nephrology, Faculty of Medicine, Wroclaw Medical University, Borowska Street 213, 50-556 Wroclaw, Poland; 9 Screening of Biological Activity Assays and Collection of Biological Material Laboratory, Wroclaw Medical University Biobank, Wroclaw Medical University, Borowska Street 211A, 50-556 Wroclaw, Poland; 10 2nd Clinical Department of Gynecology and Obstetrics, Faculty of Medicine, Wroclaw Medical University, Borowska Street 213, 50-556 Wroclaw, Poland; 11 Clinical Department of Cardiology, Wroclaw Medical University, Borowska Street 213, 50-556 Wroclaw, Poland; 12 Clinical Department of Rheumatology and Internal Medicine, Faculty of Medicine, Wroclaw Medical University, Borowska Street 213, 50-556 Wroclaw, Poland; 13 Clinical Department of Emergency Medicine, Faculty of Medicine, Wroclaw Medical University, Borowska Street 213, 50-556 Wroclaw, Poland; 14 Clinical Department of Nephrology and Transplantation Medicine, Faculty of Medicine, Wroclaw Medical University, Borowska Street 213, 50-556 Wroclaw, Poland; 15 Department of Cardiology, The Copper Health Centre (MCZ), M. Sklodowskiej-Curie Street 66, 59-300 Lubin, Poland; 16 Clinical Department of Angiology, Hypertension and Diabetology, Wroclaw Medical University, Borowska Street 213, 50-556 Wroclaw, Poland; 17 Institute of Heart Diseases, Wroclaw Medical University, Borowska Street 213, 50-556 Wroclaw, Poland; 18 Institute of Heart Diseases, University Hospital in Wroclaw, Borowska Street 213, 50-556 Wroclaw, Poland

**Keywords:** COVID-19, coronary artery disease, C_2_HEST score, risk assessment, SARS-CoV-2, mortality, risk score, outcomes, predictive value, cardiac injury

## Abstract

Background: Even though coronary artery disease (CAD) is considered an independent risk factor of an unfavorable outcome of SARS-CoV-2-infection, the clinical course of COVID-19 in subjects with CAD is heterogeneous, ranging from clinically asymptomatic to fatal cases. Since the individual C_2_HEST components are similar to the COVID-19 risk factors, we evaluated its predictive value in CAD subjects. Materials and Methods: In total, 2183 patients hospitalized due to confirmed COVID-19 were enrolled onto this study consecutively. Based on past medical history, subjects were assigned to one of two of the study arms (*CAD* vs. *non-CAD*) and allocated to different risk strata, based on the C_2_HEST score. Results: The CAD cohort included 228 subjects, while the non-CAD cohort consisted of 1956 patients. *In-hospital*, *3-month* and *6-month* mortality was highest in the *high-risk* C_2_HEST stratum in the CAD cohort, reaching 43.06%, 56.25% and 65.89%, respectively, whereas in the non-CAD cohort in the *high-risk* stratum, it reached: 26.92%, 50.77% and 64.55%. Significant differences in mortality between the C_2_HEST stratum in the CAD arm were observed in post hoc analysis only for *medium-* vs. high-risk strata. The C_2_HEST score in the CAD cohort could predict hypovolemic shock, pneumonia and acute heart failure during hospitalization, whereas in the non-CAD cohort, it could predict cardiovascular events (myocardial injury, acute heart failure, myocardial infract, carcinogenic shock), pneumonia, acute liver dysfunction and renal injury as well as bleedings. Conclusions: The C_2_HEST score is a simple, easy-to-apply tool which might be useful in risk stratification, preferably in non-CAD subjects admitted to hospital due to COVID-19.

## 1. Introduction

In March 2020, the World Health Organization (WHO) declared that coronavirus disease 2019 (COVID-19), caused by the severe acute respiratory syndrome coronavirus 2 (SARS-CoV-2), had become a global pandemic. Since the outbreak, COVID-19 has had an undeniable impact on most healthcare systems around the world and put them under unprecedented pressure. In the face of limited resources, considering that clinical manifestations of COVID-19 remain unclear—with a variety of presentations, from asymptomatic disease to lethal acute respiratory distress syndrome (ARDS) [1,2]—early identification of subjects at high risk is essential for optimizing resources and saving human lives.

During the initial phase of the pandemic, numerous risk factors (including age, male sex, chronic pulmonary diseases, diabetes and cardiovascular disorders) for COVID progression were identified. Coronary artery disease (CAD) has been postulated as an independent risk factor for an unfavorable outcome of the SARS-CoV-2 infection [3]. Furthermore, CAD is associated with a high burden of comorbidities, which additionally increases vulnerability to COVID-19 and obscures the relevant risk prognosis. There is an urgent unfulfilled need for developing a diagnostic tool focused on supporting the process of patient segregation in the initial stage of infection, especially in the high-risk population with a history of CAD.

The C_2_HEST score is a well-validated scoring system, initially designed to predict the development of atrial fibrillation (AF) in the all-comers population [4], or long-term clinic outcomes [5]. Considering that individual components of the C_2_HEST score (coronary artery disease, chronic obstructive pulmonary disease, hypertension, advanced age, chronic heart failure and thyroid disease) are the risk factors for unfavorable COVID-19 clinical course [6], and given that the C_2_HEST score can predict adverse outcomes in SARS-CoV-2 positive individuals [7,8,9,10], we assumed that it could predict clinical outcomes among subjects with CAD suffering from COVID-19.

## 2. Materials and Methods

### 2.1. Study Design and Population

This study was a part of the COronavirus in LOwer Silesia study (COLOS). The COLOS research project is an observational, retrospective registry of patients with COVID-19 hospitalized in the University Hospital in Wroclaw between February 2020 and June 2021. The study received local ethics committee approval (No. KB-444/2021), and due to legal restrictions (retrospective, observational nature), written informed consent of participation was waived.

Anonymized medical data of 2184 consecutive patients with an initial diagnosis of COVID-19 (confirmed with a reverse transcription-polymerase chain reaction (RT-PCR) for viral RNA of nasopharyngeal swab specimens) admitted to the University COVID Center between February 2020 and June 2021 were collected. From all the pre-screened cases, we carefully selected 228 subjects with confirmed CAD (past medical history of myocardial infarction (MI) and/or percutaneous coronary intervention (PCI) and/or coronary artery bypass graft (CABG)). This population of patients was qualified for the CAD arm of the study. Data of patients (1956) without prior diagnosis of CAD and signs/symptoms suggesting the presence of CAD were pooled into the non-CAD arm.

All patients, in both study arms (CAD and non-CAD), were allocated to the 3 separate groups depending on their C_2_HEST score. The C_2_HEST score value was defined by using data from past medical history and interviews.

The C_2_HEST score was calculated according to six different variables: coronary artery disease (1 point), chronic obstructive pulmonary disease (1 point each), hypertension (1 point), elderly (age ≥75 years, 2 points), systolic HF (2 points) and thyroid disease (1 point). All subjects were assigned to separate groups regarding the calculation result—the low-risk stratum of 0 or 1 point, the medium-risk stratum of 2 or 3 points, and the high-risk stratum of 4 or more points.

### 2.2. Study Outcomes

The primary outcome of this study was all-cause mortality. The data regarding death were collected in the in-hospital period, 3 months after discharge and 6 months after index hospitalization.

Additional, non-fatal outcomes included the need for mechanical ventilation support, development of pneumonia, pulmonary embolism, acute heart failure, myocardial injury, stroke, acute kidney injury, acute liver dysfunction, active bleedings, shock, multiple organ dysfunction syndrome (MODS), systemic inflammatory response syndrome (SIRS), sepsis and also the data regarding end of hospitalization (discharge home, transfer to another hospital because of deterioration and need for post-COVID-19 ambulatory rehabilitation). All the secondary outcomes were monitored during the whole hospitalization period and 6 months following hospitalization.

### 2.3. Statistical Analysis

The statistical analysis was performed by trained Medical Statisticians with R version 4.0.4 using packages time-ROC, pROC [11], survival [12], coin [13] and odds ratio [14]. A significance level of 0.05 was selected for all statistical analyses.

Descriptive data are presented as numbers and percentages for categorical variables, and as mean with standard deviation range (minimum–maximum) and number of non-missing values for numerical variables. As omnibus test, chi-square test was used for categorical variables with more than 5 expected cases in each group, whereas Fisher’s exact test was used for cases with lower cell counts. Welch’s ANOVA was performed for continuous variables due to unequal variances between risk strata and sample size large enough for appropriateness of asymptotic results. Post hoc analysis for continuous variables was performed using the Games–Howell test with Tukey correction. For categorical variables, the post hoc test was the same as the omnibus test but performed in subgroups with Bonferroni correction.

In-hospital mortality and all-cause mortality were available as right-censored data, so time-dependent ROC analysis with inverse probability of censoring weighting (IPCW) estimation was performed for those variables. The C_2_HEST score was assessed through the time-dependent area under the curve (AUC). Log-rank test was used to confirm differences in survival curves between risk strata. Proportional hazard assumption was verified using the Grambsch–Therneau test. A Cox proportional-hazards model was used to analyze the hazard ratio (HR) for C_2_HEST score, its components and risk strata.

For secondary outcomes, due to their dichotomic nature, a logistic regression model was fitted. Classical ROC analysis was performed, and AUC measurement was used for assessing predictive capabilities. Odds ratio (OR) was reported as effect size for influence of C_2_HEST score, its components and risk strata.

## 3. Results

### 3.1. Study Population Characteristics and Clinical Features

The first study group was composed of 228 subjects with confirmed significant CAD, allocated to three C_2_HEST risk strata: low risk with 12 subjects, medium risk 72 patients and high risk 144 participants. After initial allocation, the non-CAD group was composed of 1955 subjects. In this study arm, the most numerous C_2_HEST risk stratum was low risk with 1405 participants; medium risk consisted of 420 patients and the high-risk stratum comprised 130 subjects.

The baseline characteristics of both study arms are summarized in Table 1. In both study groups, higher C_2_HEST risk stratum was associated with more advanced age of subjects, along with a higher prevalence of comorbidities. This was observed in both study groups regarding the following comorbidities: hypertension, atrial fibrillation/flutter, heart failure, moderate-to-severe valvular heart disease, peripheral artery diseases and chronic kidney disease (COPD). The relationship of the C_2_HEST risk stratum with the prevalence of cardiovascular disorders (such as diabetes mellitus, history of myocardial infarction and stroke/TIA, history of thyroid disorders) was observed only in the non-CAD arm.

The differentiation between C_2_HEST risk groups in terms of therapy applied before admission to hospital concerned almost all classes of considered drugs in the non-CAD arm. However, among patients with coronary artery diseases, statistically, significant differences were observed only between low-risk and high-risk strata regarding the use of angiotensin-converting enzyme inhibitors (ACEI), beta blockers and oral antidiabetics other than sodium-glucose co-transporter-2 inhibitors (SGLT2-inhibitors) and metformin. All data relating to the treatment applied prior to the index hospitalization are presented in Table 2.

Abnormalities during an initial physical examination in the CAD cohort differences among C_2_HEST strata were examined for diastolic blood pressure and wheezing. On the other hand, in the non-CAD arm, significant differences among different C_2_HEST strata were observed in initial physical examination findings (crackles, wheezing, pulmonary congestion and peripheral edema), vital signs—systolic blood pressure, blood oxygen saturation (SpO2 on room air), and patient-reported symptoms (cough, dyspnoea and smell dysfunction). Table 3 collects patient-reported symptoms, vital signs and abnormalities measured during physical examination in both study groups.

The detailed characteristics of the laboratory assay measured at the time of admission and discharge from hospital in both studied cohorts are presented in Table 4. Regarding the complete blood count parameters, statistical differences among the C_2_HEST strata were observed only in admission level of hemoglobin (in the higher C_2_HEST stratum, a lower mean level of hemoglobin was measured) in the non-CAD cohort. On the other hand, in terms of arterial blood gases (ABG) and acid–base balance parameters, significant differences between low-risk and high-risk C_2_HEST subjects were noticed for PaO_2_ and PaCO_2_ but only in the CAD cohort. Interestingly, no significant differences between groups in terms of initial inflammatory parameters (CRP, procalcitonin, IL-6) along with liver function markers (AST, ALT) were noted. In both study cohorts with an increase in the C_2_HEST score value, we observed a rising level of cardiac injury markers (troponin, BNP). When we analyzed parameters of renal function, a high C_2_HEST score correlated with high creatinine, urea level and decreased eGFR level at admission and discharge time in the non-CAD cohort, while in the CAD cohort, this relationship was noticed only at the time of discharge.

### 3.2. Applied Treatment

In the CAD cohort, no significant differences in pharmacological management between the C_2_HEST strata were observed. However, in the non-CAD cohort subjects in the low-risk stratum were more prone to receive convalescent plasma and less often antibiotics (Table 5).

The need for oxygen supplementation increased with the C_2_HEST score, including the high-flow nasal cannula and the invasive ventilation, in the non–CAD cohort. Consequently, the oxygenation parameters from the period of qualification for advanced respiratory support decreased. On the other hand, in the CAD’s arm, no significant differences in oxygen supplementation occurred. Analogous no differences in catecholamine support, hemodialysis, and coronary revascularization procedure, in both study groups (Table 6.).

### 3.3. Association of C_2_HEST Score Results with Mortality

#### 3.3.1. Mortality

The in-hospital, 3-month and 6-month mortality was highest in the high-risk C_2_HEST stratum in the CAD cohort, reaching 43.06%, 56.25% and 65.89%, respectively, and in the non-CAD cohort, in the high-risk stratum reached 26.92%, 50.77% and 64.55%, respectively. Statistically significant differences between C_2_HEST strata in the CAD arm were observed in the post hoc analysis only when comparing the medium vs. high-risk strata. For comparison, in the non-CAD cohort, post hoc analysis revealed significant differences between low-risk and medium-risk, and low-risk and high-risk C_2_HEST strata. All the data regarding mortality in both study groups are presented in Table 7.

#### 3.3.2. Discriminatory Performance of the C_2_HEST Score for Total All-Cause Mortality

**Figure 1:**
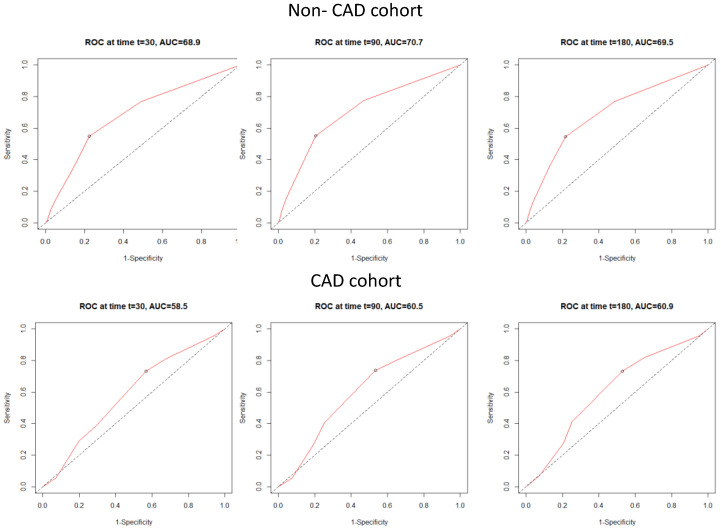
Time-dependent receiver operating characteristic (ROC) curves for all-cause mortality in both study cohorts.

The receiver operating characteristic (ROC) curves revealed that C_2_HEST poorly predicts 1-, 3- and 6-month mortality in the CAD cohort. However, the C_2_HEST score revealed higher sensitivity in the non-CAD group. The AUCs for C_2_HEST predictive value for all-cause mortality in the CAD and non-CAD cohorts are presented in Figure 1. The 1-month AUC = 58.5 vs. 68.9%; 3-month AUC = 60.5 vs. 70.7%; 6-month AUC = 60.9 vs. 69.5%.

**Figure 2:**
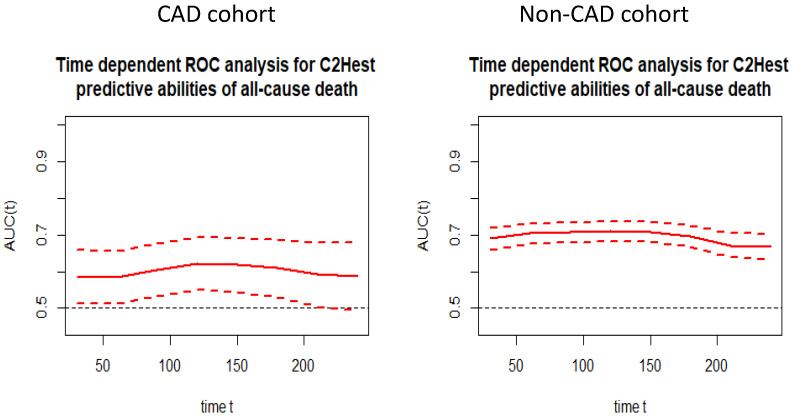
Time-dependent ROC analysis for the C_2_HEST predictive abilities of all-cause death in both study cohorts.

In the next step, we performed the time-ROC analysis in order to assess the predictive value of C_2_HEST scale for all-cause mortality at a particular time from admission to hospital in both cohorts. Figure 2 presents the time-dependent changes in predictive value of the C_2_HEST score.

**Figure 3:**
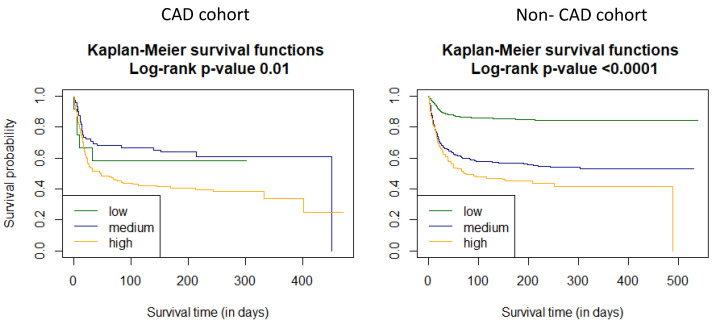
Survival curves for the C_2_HEST strata in both study cohorts estimated by Kaplan–Meier function.

Additionally, we used Kaplan–Meier functions to estimate the survival curves for the C_2_HEST strata in both study cohorts (Figure 3).

Analysis based on the Cox models was performed to assess the effect of the C_2_HEST score stratification on COVID-19 mortality. The overall model takes an uncategorized value of the C_2_HEST score, and the assumption of proportional hazard was met. An additional point in the C_2_HEST score increased the total-death intensity in approximately 47.6% of the non-CAD cohort subjects (HR 1.4764, 95% CI 1.404–1.553 *p* < 0.0001). However, in the CAD cohort, the results did not reach statistical significance. Furthermore, considering the categorized model, the change in the CAD cohort while transferring from the low-risk stratum to the high-risk stratum resulted in an increased all-cause death intensity of 4.847 times (Table 8). Interestingly, in the non-CAD cohort, switching from the low-risk to the medium-risk stratum and from the low-risk to the high-risk stratum did not achieve statistical significance (Table 9).

Table 10 and Table 11 present the association level between individual C_2_HEST score components and mortality in both study cohorts. The highest prognostic value for all-cause- death in both study groups was recorded for age (in CAD arm 1776 vs. 2946 in non-CAD cohort).

As in the previous analysis, it has been shown that age was among the most important and significant components of the C_2_HEST score in terms of survival analysis (in the Cox model), for which a bifactorial Cox model was built. The analysis of the influence of age in the CAD and non-CAD groups on all-cause mortality as well as the predictive value of C_2_HEST components on the endpoints in the CAD and non-CAD groups are presented in the Supplementary Materials.

Subsequently, we verified whether the original C_2_HEST score risk groups are the best possible stratification system. Due to the difference in Kaplan–Meier survival curves, all the possible C_2_HEST intervals were analyzed in both study cohorts, and for each, we calculated the log-rank statistics (Table 12 and Table 13). The results suggest that in both study subpopulations, the original C_2_HEST-score risk strata were not best matched. In the CAD cohort, the highest value of the log-rank corresponded with risk strata estimated as follows: 0–3 low, 4 medium and 5–8 high. On the other hand, in the non-CAD cohort, the highest value was achieved for 0 low, 1 medium and 2–8 high.

### 3.4. Association of C_2_HEST Score Results with Non-Fatal Clinical Outcomes

**Figure 4:**
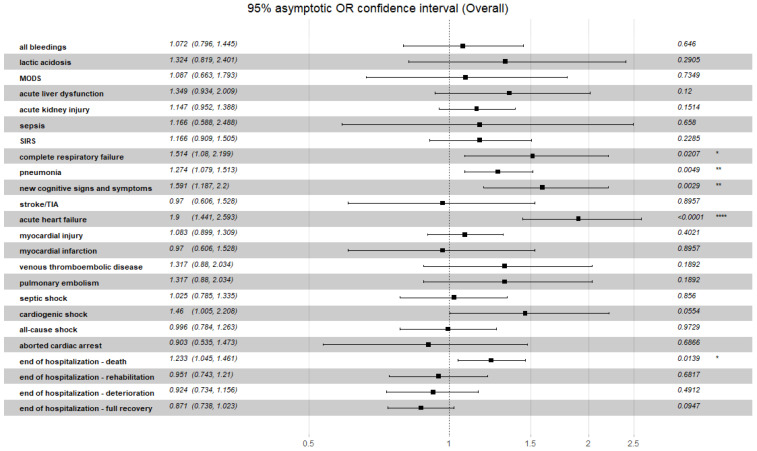
Overall odds ratio for the discriminatory performance of the C_2_HEST score for clinical non-fatal events in CAD cohort. *p*-value: Significance code. **** *p* < 0.0001, ** *p* < 0.01, * *p* < 0.05.

**Figure 5:**
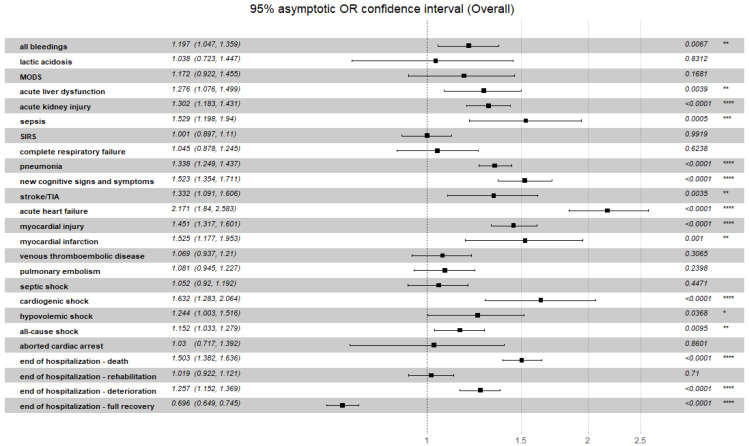
Overall odds ratio for the discriminatory performance of the C_2_HEST score for clinical non-fatal events in non-CAD cohort. *p*-value: Significance code. **** *p* < 0.0001, *** *p* < 0.001, ** *p* < 0.01, * *p* < 0.05.

A summarized discriminatory performance of the C_2_HEST score for non-fatal clinical outcomes is presented in Table 14. The significant correlation between C_2_HEST risk score in the CAD cohort and prevalence of clinical events occurred for the duration of hospitalization: hypovolemic shock, pneumonia and acute heart failure. In the non-CAD cohort, in terms of non-fatal events, significant differences between the C_2_HEST risk score strata were observed for cardiovascular events (myocardial injury, acute heart failure, myocardial infarction, cardiogenic shock), pneumonia, acute liver dysfunction and renal injury along with bleedings. Additionally, the overall odds ratio for the discriminatory performance of the C_2_HEST score for clinical non-fatal events is presented in Figure 4 (CAD) and Figure 5 (non-CAD).

## 4. Discussion

This is the first study to show the usefulness of C_2_HEST score analysis in predicting outcomes in subjects hospitalized due to SARS-CoV-2 infection with concomitant coronary artery disease vs. outcomes in those without documented CAD.

Predominantly, the initial clinical manifestations of COVID- 19 are related to the respiratory system. However, with ongoing infection, severe cardiovascular damage can occur, affecting mortality [15]. In addition, coronary artery disease is one of the major risk factors for an unfavorable outcome of SARS-CoV-2 infection. Furthermore, the prevalence of CAD in subjects hospitalized with COVID-19 is higher compared to the general population [16]. Possible explanations for the higher incidence in COVID-19 patients with CAD include comorbidity with co-occurring senescence, resulting in decreased immune system function predisposition to severe course of the disease [17,18].

The dynamics of the infection and probability of complications rate are unpredictable even in patients with a strong risk factor such as CAD, which often co-occurs with other risk factors—diabetes mellitus, hypertension arterials, male gender. Due to limited resources, there is a strong need for developing a simple, fast triage tool focused on selecting the most vulnerable patients, particularly in a high-risk cohort of patients with “classical” risk factors of unfavorable outcomes such as CAD during admission time. Several laboratory features [19] along with some recently proposed novel prognostic scales [20] have been postulated to predict clinical outcomes in patients with COVID-19 at the time of hospital admission.

However, most of them have a high level of complexity, not allowing for their smooth implementation in clinical practice. Moreover, data dedicated to subjects with CAD are missing. Given that the results of our previous study suggest that the C_2_HEST score has a good ability to predict adverse outcomes in the general population of COVID-19 patients, we decided to re-evaluate this scale in patients with CAD.

Analysis of the prevalence of all-cause death in both cohorts revealed significant differences between the C_2_HEST score stratum. However, in the CAD arm, statistically significant differences were noticed only between medium-risk and high-risk strata. Moreover, the time-ROC analysis confirmed the poor predictive value of the C_2_HEST scale for all-cause mortality in the CAD cohort. Furthermore, the Cox-model-based analysis did not reach statistical significance in predicting COVID-19 mortality. This fact is probably related to previous reports [8,21,22], which highlight cardiovascular disease as a strong independent risk factor of mortality in COVID-19. Therefore, CAD probably abolished the resolving power of the C_2_HEST score in this subpopulation, which is reflected in the poor discriminatory performance of the C_2_HEST score in terms of six-month survival probability evaluated in survival curves. Similarly, we combined high mortality in the high-risk stratum in both study arms with coexisting significantly higher mortality in the CAD cohort in low- and medium-risk strata. Additionally, the better predictive value of the C_2_HEST score in the non-CAD cohort suggests that CAD per se determines high COVID-19 mortality, as well as in the post-hospital follow-up period. Furthermore, outcomes of the log-rank statistics performed in the CAD cohort suggest that other comorbidities have a lower impact on the predictive value of the C_2_HEST score.

Similar observations (higher mortality rate, lower differences in predictive value of individual C_2_HEST score stratum) had been previously described in the diabetic COVID-19 population [8]. It needs to be emphasized that not treating coronary artery diseases in CAD-related patients is an independent risk factor [23].

Analysis of the non-fatal secondary outcome provides other interesting findings: in the CAD cohort, the C_2_HEST score had a predictive value only in terms of acute heart failure and hypovolemic shock. However, in the non-CAD cohort, it allowed for predicting cardiovascular disorders (cardiogenic shock, myocardial injury, infarct, acute heart failure episode, stroke or TIA), all types of bleedings, acute renal injury and acute liver dysfunction together with infection complications (pneumonia sepsis). Considering that, in this cohort, there were no significant differences between the C_2_HEST strata in terms of laboratory assays considered as “classic markers of severity”—CRP, leucocytes level, procalcitonin, PaO_2_ and LDH—we can assume that C_2_HEST score might be a valuable triage tool in this subpopulation of COVID-19 patients. When we compile the obtained results and compare them with other studies focused on the evaluation of a C_2_HEST score in subjects with COVID 19 [5,6,7,8], we can conclude that C_2_HEST has the highest predictive value in patients without a high burden of cardio-metabolic disorders. On the other hand, combined results confirm that cardiovascular disorders are a strong independent risk factor of mortality in C_2_HEST score and can provide a foundation for creating a new predictive scale for COVID-19 subjects’ outcomes.

Several scales focused on predicting unfavorable COVID-19 outcomes have been introduced to clinical practice [24,25,26]. However, most of them were based on numerous clinical data, limiting their implementation as a triage tool. Concerning previous [5,6,7,8] along with presented results, it might be advisable to combine the C_2_HEST score with other mentioned predictions in selected subpopulations (diabetes and/or CAD) to achieve a triage tool with better discriminatory performance. This concept might be useful in high-risk COVID-19 subpopulations; however, future evaluation is required.

## 5. Conclusions

The present study is the first demonstration of the differences between patients with and without coronary artery diseases in the predictive value of the C_2_HEST score in subjects hospitalized due to COVID-19. The C_2_HEST score in the non-CAD cohort showed a better performance in terms of prediction of adverse outcomes in both in-hospital and 6-month-mortality and other non-fatal clinical outcomes such as cardiovascular events (myocardial injury, acute heart failure, myocardial infraction, carcinogenic shock), pneumonia, acute liver dysfunction and renal injury along with bleedings. The C_2_HEST-score is a simple, easy-to-apply tool which might be useful in risk stratification in CAD and non-CAD subjects admitted to hospital due to COVID-19.

## 6. Limitations

This study has several limitations. First, the retrospective structure along with single-center evaluation could partially affect the results. Additionally, data collection and clinical management were performed despite limited resources, which might have a negative impact on the outcomes. Finally, some clinical data and baseline laboratory assays are incomplete, hindering appropriate interpretation of the results.

## Figures and Tables

**Table 1:** Baseline characteristics after C_2_HEST risk stratification in the CAD and non-CAD study cohorts.

Variables, Units (N) (CAD/non-CAD)	Low Risk (0–1)		Medium Risk (2–3)		High Risk (≥4)		OMNIBUS *p* Value		*p* Value (For Post Hoc Analysis)	
	Min–Max (N) or n/N (% of Risk Category)		Min–Max (N) or n/N (% of Risk Category)		Min–Max (N) or n/N (% of Risk Category)					
	CAD	Non- CAD	CAD	Non- CAD	CAD	Non- CAD	CAD	Non- CAD	CAD	Non- CAD
Demographics										
**Age, years** (228/1955)	67.92 ± 5.45 59–73 (12)	50.96 ± 15.88 17–74 (1405)	66.94 ± 6.95 49–84 (72)	77.03 ± 11.77 29–100 (420)	75.9 ± 9.65 38–93 (144)	81.56 ± 8.11 43–100 (130)	<0.0001	<0.0001	0.849 ^a^ 0.0008 ^b^ <0.0001 ^c^	<0.0001 ^a,b,c^
**Male gender** (228/1955)	11/12 (91.67%)	724/1405 (51.53%)	52/72 (72.22)	156/420 (37.14%)	98/144 (68.06%)	41/130 (31.54%)	0.2188	<0.0001	N/A	<0.0001 ^a,b^ 0.8675 ^c^
**BMI kg/m^2^** (66/488)	29.43 ± 6.99 24.49–34.37 (2)	28.27 ± 5.07 15.36–49.38 (395)	28.85 ± 4.14 22.6–35.16 (19)	29.4 ± 5.98 18.59–47.75 (71)	27.78 ± 4.88 16.41–38.97 (45)	27.81 ± 7.59 17.28–48.21 (22)	0.738	0.3172	N/A	N/A
**Obesity** **(BMI ≥30 kg/m^2^)** (66/488)	1/2 (50.0%)	131/395 (33.17%)	9/19 (47.37%)	29/71 (40.85%)	15/45 (33.33)	6/22 (27.27%)	0.805	0.4104	N/A	N/A
**Cigarette smoking** **never** **previous** **current** (228/1955)	12/12 (100%) 0/12 (0%) 0/12(0%)	1325/1405 (94.31%)46/1405 (3.27%) 34/1405 (2.42%)	58/72 (81.69%) 6/72 (8.45%) 7/72 (9.86%)	373/420 (89.23%) 29/420 (6.94%) 16/420 (3.83%)	105/144 (72.92%) 24/144 (16.67%) 15/144 (10.42%)	113/130 (87.6%) 12/130 (9.3%) 4/130 (3.1%)	0.2136	0.0005	N/A	0.0049 ^a^ 0.0138 ^b^ 1.0 ^c^
**Comorbidities**										
**Hypertension** (228/1955)	0/12 (0%)	415/1405 (29.54%)	58/72 (80.56%)	299/420 (71.19%)	131/144 (90.97)	118/130 (90.77%)	<0.0001	<0.0001	<0.0001 ^a,b^ 0.1424 ^c^	<0.0001 ^a,b,c^
**DM** (228/1955)	3/12 (25.0%)	205/1405 (14.6%)	31/72 (43.06%)	115/420 (27.39%)	68/144 (47.22%)	50/130 (38.46%)	0.4607	<0.0001	N/A	<0.0001 ^a,b^ 0.2594 ^c^
**Atrial fibrillation/flutter** (228/1955)	1/12 (8.33%)	48/1405 (3.42%)	15/72 (20.83%)	91/420 (21.67%)	73/144 (50.69%)	62/130 (47.69%)	<0.0001	<0.0001	1.0 ^a^ 0.0158 ^b^ <0.0001 ^c^	<0.0001 ^a,b,c^
**Previous coronary revascularization** (228/1955)	6/12 (50%)	0/1405 (0%)	37/72 (51.39%)	0/420 (0%)	111/144 (77.08%)	0/130 (0%)	0.0002	<0.0001	1.0 ^a^ 0.2238 ^b^ 0.0005 ^c^	<0.0001 ^a,b,c^
**Previous myocardial infarction** (228/1955)	11/12 (91.67%)	0/1405 (0%)	63/72 (87.5%)	0/420 (0%)	117/144 (81.25%)	0/130 (0%)	0.4723	<0.0001	N/A	<0.0001 ^a,b,c^
**Heart failure** (228/1955)	0/12 (0%)	0/1405 (0%)	6/72 (8.33%)	47/420 (11.19%)	112/144 (77.78%)	90/130 (69.23%)	<0.0001	<0.0001	1.0 ^a^ <0.0001 ^b,c^	<0.0001 ^a,b,c^
**Moderate/severe valvular heart disease** **or previous valve heart surgery** (228/1955)	0/12 (0%)	13/1405 (0.93%)	6/72 (8.33%)	26/420 (6.19%)	29/144 (20.14)	22.130 (16.92%)	0.0227	<0.0001	1.0 ^a^ 0.3738 ^b^ 0.0924 ^c^	<0.0001 ^a,b^ 0.0016 ^c^
**Peripheral artery disease** (228/1955)	2/12 (16.67%)	24/1405 (1.71%)	6/72 (8.33%)	25/420 (5.95%)	35/144 (24.31%)	8/130 (6.15%)	0.0113	<0.0001	0.9599 ^a^ 1.0 ^b^ 0.0159 ^c^	<0.0001 ^a^ 0.0118 ^b^ 1.0 ^c^
**Previous stroke/TIA** (228/1955)	1/12 (8.33%)	46/1405 (3.27%)	11/72 (15.28%)	48/420 (11.43%)	37/144 (25.69%)	21/130 (16.15%)	0.1331	<0.0001	N/A	<0.0001 ^a,b^ 0.6124 ^c^
**Chronic kidney disease** (228/1955)	0/12 (0%)	70/1405 (4.98%)	16/72 (22.22%)	54/420 (12.86%)	54/144 (37.5%)	37/130 (28.46%)	0.0022	<0.0001	0.3325 ^a^ 0.0256 ^b^ 0.0915 ^c^	<0.0001 ^a,b^ 0.0002 ^c^
**Haemodialysis** (228/1955)	0/12 (0%)	19/1405 (1.35%)	8/72 (11.11%)	12/420 (2.86%)	15/144 (10.42%)	4/130 (3.08%)	0.7478	0.0488	N/A	0.1505 ^a^ 0.3709 ^b^ 1.0 ^c^
**Asthma** (228/1955)	0/12 (0%)	54/1405 (3.84%)	2/72 (2.78%)	18/420 (4.29%)	6/144 (4.17%)	3/130 (3.85%)	0.8207	0.9184	N/A	N/A
**COPD** (228/1955)	0/12 (0%)	6/1405 (0.43%)	3/72 (4.17%)	22/420 (5.24%)	21/144 (14.58%)	23/130 (17.69%)	0.0317	<0.0001	1.0 ^a^ 1.0 ^b^ 0.0657 ^c^	<0.0001 ^a,b,c^
**Hypothyroidism** (228/1955)	0/12 (0%)	76/1405 (5.41%)	6/72 (8.33%)	62/420 (14.76%)	22/144 (15.28%)	42/130 (32.31%)	0.4089	<0.0001	N/A	<0.0001 ^a,b^ 0.0003 ^c^
**Hyperthyroidism** (228/1955)	0/12 (0%)	4/1405 (0.28%)	2/72 (2.78%)	8/420 (1.9%)	5/144 (3.47%)	2/130 (1.54%)				

Continuous variables are presented as: mean ± SD, range (minimum–maximum) and number of non-missing values. Categorized variables are presented as a number with a percentage. Information about the numbers with valid values is provided in the left column. Abbreviations: CAD—coronary artery disease, OMNIBUS—analysis of variance, N—valid measurements, n—number of patients with parameter above cut-off point, SD—standard deviation, BMI—body mass index, DM—diabetes mellitus, TIA—transient ischemic attack, COPD—chronic obstructive pulmonary disease, N/A—non-applicable, a—low risk vs. medium risk, b—low risk vs. high risk, c—medium risk vs. high risk.

**Table 2:** Baseline characteristics of the study cohort—treatment applied before hospitalization.

Variables, Units (N) (CAD/non-CAD)	Low Risk (0–1)		Medium Risk (2–3)		High Risk (≥4)		OMNIBUS *p* Value		*p* Value (For Post Hoc Analysis)	
	n/N (% of Risk Category)		n/N (% of Risk Category)		n/N (% of Risk Category)					
	CAD	Non-CAD	CAD	Non-CAD	CAD	Non-CAD	CAD	Non-CAD	CAD	Non-CAD
Treatment Applied before Hospitalization										
**ACEI** (228/1955)	1/12 (8.33%)	115/1405 (8.19%)	29/72 (40.28%)	91/420 (21.67%)	73/144 (50.69%)	43/130 (33.08%)	0.0109	<0.0001	0.2096 ^a^ 0.0349 ^b^ 0.5797 ^c^	<0.0001 ^a,b^ 0.0341 ^c^
**ARB** (228/1955)	0/12 (0%)	76/1405 (5.41%)	6/72 (8.33%)	232/420 (7.62%)	13/144 (9.03%)	17/130 (13.08%)	0.7769	0.0015	N/A	0.3519 ^a^ 0.0028 ^b^ 0.2494 ^c^
**MRA** (228/1955)	0/12 (0%)	18/1405 (1.28%)	9/72 (12.5%)	24/420 (5.71%)	29/144 (20.14%)	20/130 (15.38%)	0.113	<0.0001	N/A	<0.0001 ^a,b^ 0.0038 ^c^
**β-blocker** (228/1955)	3/12 (25.0%)	194/1405 (13.81%)	42/72 (58.33%)	137/420 (32.62%)	94/144 (65.28%)	63/130 (48.46%)	0.0203	<0.0001	0.1727 ^a^ 0.0308 ^b^ 1.0 ^c^	<0.0001 ^a,b^ 0.0045 ^c^
**Digitalis glycoside** (228/1955)	0/12 (0%)	3/1405 (0.21%)	1/72 (1.39%)	5/420 (1.19%)	4/144 (2.78%)	6/130 (4.62%)	0.7464	<0.0001	N/A	0.0568 ^a^ <0.0001 ^b^ 0.0752
**Calcium channel blocker** **(non-** **dihydropyridines)** (228/1955)	0/12 (0%)	11/1405 (0.78%)	2/72 (2.78%)	11/420 (2.62%)	5/144 (3.47%)	9/130 (6.92%)	1.0	<0.0001	N/A	0.0238 ^a^ <0.0001 ^b^ 0.924 ^c^
**Calcium channel blocker** **(dihydropyridines)** (228/1955)	0/12 (0%)	103/1405 (7.33%)	17/72 (23.61%)	67/420 (15.95%)	41/144 (28.47%)	33/130 (25.38%)	0.0707	<0.0001	N/A	<0.0001 ^a,b^ 0.0632 ^c^
**α-adrenergic blocker** (228/1955)	0/12 (0%)	45/1405 (3.20%)	9/72 (12.5%)	25/420 (5.95%)	23/144 (15.97%)	16/130 (12.31%)	0.3087	<0.0001	N/A	0.453 ^a^ <0.0001 ^b^ 0.0793 ^c^
**Thiazide or** **thiazide-like** **diuretic** (228/1955)	0/12 (0%)	68/1405 (4.84%)	4/72 (5.56%)	43/420 (10.24%)	20/144 (13.89%)	15/130 (11.54%)	0.0915	<0.0001	N/A	0.0003 ^a^ 0.0074 ^b^ 1.0 ^c^
**Loop diuretic** (228/1955)	0/12 (0%)	39/1405 (2.78%)	13/72 (18.06%)	52/420 (12.38%)	48/144 (33.33%)	33/130 (25.38%)	0.0038	<0.0001	0.5945 ^a^ 0.0554 ^b^ 0.0729 ^c^	<0.0001 ^a,b^ 0.0017 ^c^
**Statin** (228/1955)	3/12 (25.0%)	100/1405 (7.12%)	39/72 (54.17%)	82/420 (19.52%)	88/144 (61.11%)	38/130 (29.23%)	0.0441	<0.0001	0.357 ^a^ 0.0988 ^b^ 1.0 ^c^	<0.0001 ^a,b^ 0.0792 ^c^
**Acetylsalicylic acid** (228/1955)	2/12 (16.67)	79/1405 (5.62%)	37/72 (51.39%)	58/420 (13.81%)	56/144 (38.89%)	26/130 (20.0%)	0.0419	<0.0001 ^a^	0.1645 ^a^ 0.668 ^b^ 0.3266 ^c^	<0.0001 ^a,b^ 0.3457 ^c^
**LMWH** (228/1955)	3/12 (25%)	71/1405 (5.05%)	5/72 (6.94%)	36/420 (8.57%)	19/144 (13.19%)	7/130 (5.38%)	0.1309	0.0255	N/A	0.0301 ^a^ 1.0 ^b^ 0.958 ^c^
**VKA** (228/1955)	0/12 (0%)	10/1405 (0.71%	3/72 (4.17%)	11/420 (2.62%)	11/144 (7.64%)	12/130 (9.23%)	0.5573	<0.0001	N/A	0.0092 ^a^ <0.0001 ^b^ 0.0121 ^c^
**NOAC** (228/1955)	0/12 (0%)	18/1405 (1.28%)	7/72 (9.72%)	30/420 (7.14%)	33/144 (22.92%)	19/130 (14.62%)	0.013	<0.0001	1.0 ^a^ 0.2151 ^b^ 0.0743 ^c^	<0.0001 ^a,b^ 0.0391 ^c^
**Insulin** (228/1955)	1/12 (8.33%)	61/1405 (4.34%)	6/72 (8.33%)	23/420 (5.48%)	26/144 (18.06%)	14/130 (10.77%)	0.1454	0.0051	N/A	1.0 ^a^ 0.0071 ^b^ 0.1703 ^c^
**Metformin** (228/1955)	2/12 (16.67%)	102/1405 (7.26%)	15/72 (20.83%)	52/420 (12.38%)	26/144 (18.06%)	25/130 (19.23%)	0.9147	<0.0001	N/A	0.0039 ^a^ <0.0001 ^b^ 0.2052 ^c^
**SGLT2 inhibitor** (228/1955)	0/12 (0%)	11/1405 (0.78%)	2/72 (2.78%)	5/420 (1.19%)	5/144 (3.48%)	4/130 (3.08%)	1.0	0.0446	N/A	1.0 ^a^ 0.0964 ^b^ 0.678 ^c^
**Oral antidiabetics other than SGLT2 inh. and metformin** (228/1955)	0/12 (0%)	27/1405 (1.92%)	9/72 (12.5%)	25/420 (5.95%)	17/144 (11.81%)	11/130 (8.46%)	0.5948	<0.0001	0.1727 ^a^ 0.0308 ^b^ 1.0 ^c^	0.0002 ^a^ 0.0005 ^b^ 0.9411 ^c^
**Proton pump inhibitor** (228/1955)	3/12 (25.0%)	86/1405 (6.12%)	22/72 (30.56%)	53/420 (12.62%)	51/144 (35.42%)	35/130 (26.92%)	0.676	<0.0001	N/A	<0.0001 ^a,b^ 0.0005 ^c^
**Oral corticosteroid** (228/1955)	0/12 (0%)	62/1405 (4.41%)	5/72 (6.94%)	16/420 (4.52%)	5/144 (3.47%)	1/130 (0.77%)	0.429	0.1303	N/A	N/A
**Immunosuppression other than corticosteroid** (228/1955)	0/12 (0%)	49/1405 (3.49%)	10/72 (13.89%)	12/420 (2.86%)	2/144 (1.39%)	0/130 (0%)	0.0007	0.0502	1.0 ^a^ 1.0 ^b^ 0.0011 ^c^	0.291 ^a^ 0.0836 ^b^ 0.00753 ^c^

Categorized variables are presented as a number with a percentage. Information about the numbers with valid values is provided in the left column. Abbreviations: CAD—coronary artery disease, OMNIBUS—analysis of variance, N—valid measurements, n—number of patients with parameter above cut-off point, ACEI—angiotensin-converting enzyme inhibitors, ARBs—angiotensin receptor blockers, MRAs—mineralocorticoid receptor antagonists, LMWH—low molecular weight heparin, VKA—vitamin K antagonists, NOAC—novel oral anticoagulants, SGLT2 inh.—sodium glucose co-transporter-2 inhibitors, N/A—non-applicable, a—low risk vs. medium risk, b—low risk vs. high risk, c—medium risk vs. high risk.

**Table 3:** Patient-reported symptoms, vital signs and abnormalities measured during physical examination at hospital admission in the CAD and non-CAD study cohorts after C_2_HEST risk stratification.

Variables, Units (N) (CAD/non-CAD)	Low Risk (0–1)		Medium Risk (2–3)		High Risk (≥4)		OMNIBUS *p* Value		*p* Value (For Post Hoc Analysis)	
	Min–Max (N) or n/N (% of Risk Category)		Min–Max (N) or n/N (% of Risk Category)		Min–Max (N) or n/N (% of Risk Category)					
	CAD	Non- CAD	CAD	Non- CAD	CAD	Non- CAD	CAD	Non- CAD	CAD	Non- CAD
Patient-Reported Symptoms										
**Cough** **(228/1955)**	0/12 (0%)	455/1405 (32.38%)	20/72 (27.78%)	104/420 (24.76%)	35/144 (24.31%)	34/130 (26.15%)	0.1035	0.0066	N/A	0.0107 ^a^ 0.5212 ^b^ 1.0 ^c^
**Dyspnoea** **(228/1955)**	2/12 (16.67%)	567/1405 0.36%)	29/72 (40.28%)	177/420 (42.14%)	76/144 (52.78%)	70/130 (53.85%)	0.0216	0.0113	0.6381 ^a^ 0.1063 ^b^ 0.3366 ^c^	1.0 ^a^ 0.0114 ^b^ 0.0746 ^c^
**Chest pain** **(228/1955)**	0/12 (0%)	102/1405 (7.26%)	8/72 (11.11%)	26/420 (6.19%)	17/144 (11.81%)	10/130 (7.69%)	0.6279	0.723	N/A	N/A
**Hemoptysis** **(228/1955)**	0/12 (0%)	9/1405 (0.65%)	0/72 (0%)	2/420 (0.48%)	4/144 (2.78%)	0/130 (0%)	0.44	1.0	N/A	N/A
**Smell dysfunction** **(228/1955)**	0/12 (0%)	61/1405 (4.34%)	3/72 (4.17%)	7/420 (1.67%)	4/144 (2.78%)	1/130 (0.77%)	0.7877	0.0056	N/A	0.035 ^a^ 0.1726 ^b^ 1.0 ^c^
**Taste dysfunction** **(228/1955)**	0/12 (0%)	49/1405 (3.49%)	3/72 (4.17%)	7/420 (1.67%)	4/144 (2.78%)	3/130 (2.31%)	0.7877	0.1482	N/A	N/A
**Abdominal pain** **(228/1955)**	0/12 (0%)	103/1405 (7.33%)	3/72 (4.17%)	23/420 (5.48%)	8/144 (5.56%)	9/130 (6.92%)	0.8668	0.421	N/A	N/A
**Diarrhea** **(228/1955)**	0/12 (0%)	75/1405 (5.34%)	7/72 (9.72%)	26/420 (6.19%)	7/144 (4.86%)	112/130 (9.23%)	0.3583	0.1758	N/A	N/A
**Nausea/vomiting** **(228/1955)**	0/12 (0%)	57/1405 (4.06%)	6/72 (8.33%)	21/420 (5.0%)	6/144 (4.17%)	8/130 (6.15%)	0.3055	0.4267	N/A	N/A
**Measured vital signs**										
**Body temperature** **°C** **(115/1070)**	37.8 ± 1.98 36.4–39.2 (2)	37.06 ± 0.88 34.4–40.5 (807)	36.98 ± 0.98 35.2–40.0 (41)	36.92 ± 0.9 35.0–40.0 (194)	37.03 ± 0.83 35.9–37.2 (72)	36.83 ± 0.89 35.2–40.0 (69)	0.8629	0.025	N/A	0.102 ^a^ 0.102 ^b^ 0.782 ^c^
**Heart rate** **beats/minute** **(196/1476)**	94.14 ± 23.58 70–140 (7)	86.35 ± 15.57 48–160 (1038)	82.6 ± 14.43 60–140 (60)	84.4 ± 16.83 50–160 (327)	83.79 ± 17.96 50–170 (129)	85.88 ± 19.73 36–150 (111)	0.4613	0.1793	N/A	N/A
**Respiratory rate breaths/minute** **(23/285)**	12 ± 0.0 12–12 (1)	18.38 ± 5.77 12–50 (203)	18.54 ± 4.63 12–30 (13)	18.69 ± 5.66 12–45 (55)	18.53 ± 4.25 12–30 (19)	19.85 ± 7.13 12–50 (27)	N/A	0.5836	N/A	N/A
**Systolic BP** **(196/1473)**	130.43 ± 16.3 108–150 (7)	130.72 ± 21.3 60–240 (1033)	134.63 ± 23.9 90–200 (59)	134.17 ± 25.7 50–270 (326)	133.97 ± 23.7 85–200 (130)	135.11 ± 25.9 70–210 (114)	0.8376	0.0301	N/A	0.072 ^a^ 0.192 ^b^ 0.941 ^c^
**Diastolic BP** **(196/1465)**	85.0 ± 7.35 75–97 (7)	78.5 ± 12.7 40–150 (1030)	78.83 ± 13.61 45–112 (59)	77.91 ± 13.69 40–157 (321)	75.04 ± 14.03 45–82.75 (130)	76.61 ± 16.65 40–143 (114)	0.0114	0.4284	0.19 ^a^ 0.025 ^b^ 0.188 ^c^	N/A
**SpO2 on room air, % (FiO2 = 21%)** **(129/1133)**	89.43 ± 6.8 80–99 (7)	92.87 ± 7.13 48–100 (807)	92.63 ± 4.68 81–99 (41)	89.19 ± 10.17 50–100 (240)	90.78 ± 7.92 60–99 (81)	89.6 ± 9.1 50–99 (86)	0.2165	<0.0001	N/A	<0.0001 ^a^ 0.005 ^b^ 0.935 ^c^
**Abnormalities detected during physical examination**										
**Crackles** **(228/1955)**	1/12 (8.33%)	153/1405 (10.89%)	17/72 (23.61%)	82/420 (19.52%)	37/144 (25.69%)	29/130 (22.31%)	0.4817	<0.0001	N/A	<0.000 ^a^ 0.0006 ^b^ 1.0 ^c^
**Wheezing** **(228/1955)**	1/12 (8.33%)	93/1405 (6.62%)	8/72 (11.11%)	48/420 (11.43%)	42/144 (29.17%)	27/130 (20.77%)	0.0046	<0.0001	1.0 ^a^ 0.5439 ^b^ 0.01 ^c^	0.0052 ^a^ <0.0001 ^b^ 0.0309 ^c^
**Pulmonary congestion** **(228/1955)**	1/12 (8.33%)	183/1405 (13.02%)	15/72 (20.83%)	90/420 (21.43%)	37/144 (25.69%)	41/130 (31.54%)	0.3884	<0.0001	N/A	<0.0001 ^a,b^ 0.0739 ^c^
**Peripheral edema** **(228/1955)**	1/12 (8.33%)	75/1405 (5.34%)	10/72 (13.89%)	50/420 (11.91%)	30/144 (20.83%)	23/130 (17.69%)	0.4135	<0.0001	N/A	<0.0001 ^a,b^ 0.3621 ^c^

Continuous variables are presented as: mean ± SD, range (minimum–maximum) and number of non-missing values. Categorized variables are presented as a number with a percentage. Information about the numbers with valid values is provided in the left column. Abbreviations: SD—standard deviation, CAD—coronary artery disease, OMNIBUS—analysis of variance, N—valid measurements, n—number of patients with parameter above cut-off point, SBP—systolic blood pressure, DBP—diastolic blood pressure, a—low risk vs. medium risk, b—low risk vs. high risk, c—medium risk vs. high risk.

**Table 4:** Laboratory parameters measured during hospitalization in the studied cohort.

Parameter (N) (CAD/ Non-CAD)	Time of Assessment	Units	Low Risk (0–1)		Medium Risk (2–3)		High Risk (≥4)		OMNIBUS *p* Value		*p* Value (For Post Hoc Analysis)	
			Mean ± SD Min–Ma x(N) or n/N (% of Risk Category)		Mean ± SD Min–Ma x(N) or n/N (% of Risk Category)		Mean ± SD Min–Ma x(N) or n/N (% of Risk Category)					
			CAD	Non- CAD	CAD	Non- CAD	CAD	Non- CAD	CAD	Non-CAD	CAD	Non- CAD
**Complete Blood Count (CBC)**												
**Leucocytes** (226/1822)	**On admission**	×10^3^/µL	6.93 ± 4.59 0.51–16.7 (12)	9.0 ± 12.39 0.67–304.02 (1289)	7.71 ± 3.49 1.24–20.53 (71)	9.64 ± 12.34 0.51–215.97 (409)	9.42 ± 8.97 1.83–10.91 (143)	9.12 ± 6.7 1.19–58.49 (124)	0.1152	0.6638	N/A	N/A
**Hemoglobin** (226/1822)	**On admission**	g/dL	12.25 ± 3.35 4.9–18 (12)	13.28 ± 2.13 3.9–20.3 (1289)	12.55 ± 2.43 6.8–17.0 (71)	12.57 ± 2.3 4.5–18.9 (409)	11.84 ± 2.41 5.3–18.8 (143)	12.09 ± 2.56 5.3–17.9 (124)	0.1566	<0.0001	N/A	<0.0001 ^a,b^ 0.153 ^c^
**Platelets** (226/1822)	**On admission**	×10^3^/µL	186.92 ± 8.08 58.0–326.0 (12)	236.09 ± 108.6 0–1356.0 (1289)	225.61 ± 121.3 16–735 (71)	230.85 ± 112.7 3.0–740.0 (409)	204.18 ± 81.0 8.0–243.5 (143)	232.7 ± 103.45 15.0–578 (124)	0.314	0.6934	N/A	N/A
**Acid–base balance in arterial blood gas**												
**pH** (56/220)	**On admission**		7.39 ± 0.12 7.26–7.49 (3)	7.43 ± 0.08 7.04–7.58 (118)	7.43 ± 0.05 7.35–7.51 (14)	7.43 ± 0.07 7.1–7.54 (74)	7.42 ± 0.07 7.26–7.54 (39)	7.4 ± 0.08 7.09–7.52 (28)	0.7113	0.2784	N/A	N/A
**PaO_2_** (56/220)	**On admission**		80.67 ± 2.56 78–83.1 (3)	72.04 ± 27.6 12.8–100.0 (118)	76.52 ± 19.84 45.4–100.0 (14)	76.28 ± 51.32 28.3–100.0 (74)	66.23 ± 30.97 23.7–100.0 (39)	76.68 ± 38.78 32.8–100.0 (28)	0.0316	0.7092	0.737 ^a^ 0.022 ^b^ 0.343 ^c^	N/A
**PaCO_2_** (56/220)	**On admission**		30.93 ± 2.6 28.4–33.6 (3)	38.2 ± 10.33 20.2–82.4 (118)	32.89 ± 6.05 20.9–46.5 (14)	37.37 ± 9.8 23.0–79.4 (74)	39.2 ± 11.98 19.7–88.4 (39)	37.87 ± 9.44 25.0–74.9 (28)	0.0193	0.858	0.663 ^a^ 0.014 ^b^ 0.04 ^c^	N/A
**HCO_3_^−^ standard** (56/216)	**On admission**	mmol/L	20.23 ± 4.45 15.1–23.6 (3)	25.03 ± 3.68 12.1–32.9 (117)	23.05 ± 4.08 15.9–31.8 (14)	24.63 ± 4.19 14.3–39.5 (71)	24.64 ± 4.56 17.0–38.6 (39)	23.47 ± 4.58 13.5–31.7 (28)	0.2826	0.2431	N/A	N/A
**Lactates** (53/192)	**On admission**		2.23 ± 0.85 1.6–3.2 (3)	2.46 ± 1.62 0.6–12.8 (102)	2.26 ± 0.74 1.2–3.8 (13)	21.97 ± 0.88 0.5–5.7 (66)	2.46 ± 1.37 0.8–5.9 (37)	2.6 ± 2.39 0.6–12.0 (24)	0.8139	0.0319	N/A	0.031 ^a^ 0.963 ^b^ 0.433 ^c^
**Electrolytes, inflammatory and iron biomarkers**												
**Na^+^** (225/1805)	**On admission**	mmol/L	137.17 ± 4.9 128–146 (12)	138.27 ± 4.35 106.0–159.0 (1276)	136.41 ± 4.98 121–151 (70)	137.97 ± 7.27 101.0–175.0 (405)	137.79 ± 5.59 119–163 (143)	138.08 ± 8.06 108.0–174.0 (124)	0.2145	0.709	N/A	N/A
**K^+^** (225/1812)	**On admission**	mmol/L	4.27 ± 0.62 3.29–5.7 (12)	4.06 ± 0.58 2.0–7.5 (1281)	4.14 ± 0.76 2.42 ± 7.03 (70)	4.14 ± 0.69 2.4–6.8 (407)	4.38 ± 0.89 2.97–8.7 (143)	4.19 ± 0.73 2.53–6.9 (124)	0.1606	0.0293	N/A	0.115 ^a^ 0.119 ^b^ 0.707 ^c^
**CRP** (225/1793)	**On admission**	mg/L	70.75 ± 75.51 0.32–207.32 (12)	76.52 ± 84.59 0.13–531.58 (1262)	77.9 ± 70.03 0.75–293.49 (70)	84.1 ± 89.21 0.29–538.55 (407)	80.12 ± 88.44 0.4–390.94 (143)	72.11 ± 72.35 0.4–365.22 (124)	0.9163	0.2163	N/A	N/A
	**On discharge**		92.64 ± 124.21 0.32–418.16 (12)	44.84 ± 78.56 0.13–496.98 (1262)	71.94 ± 106.15 0.22–447.61 (70)	72.72 ± 92.46 0.25–538.55 (407)	82.67 ± 92.23 0.4–431.9 (143)	63.7 ± 78.51 0.42–365.22 (124)	0.7315	<0.0001	N/A	<0.0001 ^a^ 0.084 ^b^ 0.532 ^c^
**Procalcitonin** (179/1294)	**On admission**	ng/mL	0.64 ± 0.9 0.03–2.61 (7)	0.84 ± 4.49 0.01–61.28 (911)	0.92 ± 2.59 0.02–14.84 (56)	2.01 ± 13.09 0.01–196.04 (288)	1.71 ± 5.9 0.01–49.83 (116)	1.2 ± 6.37 0.01–60.77 (95)	0.2649	0.2995	N/A	N/A
**IL-6** (68/633)	**On admission**	pg/mL	37.93 ± 16.7 20.1–53.2 (3)	61.44 ± 425.64 2.0–9099.0 (477)	73.5 ± 101.87 3.02–499 (25)	36.79 ± 50.49 2.0–398.0 (118)	69.8 ± 95.29 2.0–369 (40)	54.56 ± 96.68 2.0–421.0 (38)	0.13.02	0.2889	N/A	N/A
**D-dimer** (176/1402)	**On admission**	µg/mL	8.97 ± 23.93 0.41–80.91 (11)	3.67 ± 12.0 0.15–132.82 (991)	5.41 ± 17.27 0.29–95.86 (57)	6.69 ± 16.68 0.2–127.24 (316)	7.01 ± 21.08 0.22–128.0 (108)	3.62 ± 11.41 0.24–107.54 (95)	0.8225	0.012	N/A	0.009 ^a^ 0.999 ^b^ 0.104 ^c^
**INR** (213/1710)	**On admission**		1.09 ± 0.11 0.97–1.33 (12)	1.14 ± 0.48 0.82–15.2 (1215)	1.19 ± 0.21 0.96–1.89 (67)	1.27 ± 0.64 0.87–7.8 (378)	1.8 ± 2.59 0.9–21.1 (134)	1.75 ± 2.27 0.89–18.74 (117)	0.0017	<0.0001	0.055**^a^** 0.005**^b^** 0.02**^c^**	0.0006 ^a^ 0.011 ^b^ 0.065 ^c^
**aPTT** (207/1659)	**On admission**	>60 s	1/12 (8.33%)	27/1179 (2.29%)	0/66 (0%)	7/365 (1.92%)	6/129 (4.65%)	5/115 (4.35%)	0.0879	0.2943	N/A	N/A
**Biochemistry**												
**Glucose** (211/1547)	**On admission**	mg/dL	134.33 ± 59.96 83–307 (12)	134.85 ± 75.07 28.0–933.0 (1051)	174.7 ± 108.24 63–554 (64)	147.21 ± 28.53 47.0–1026 (385)	153.33 ± 107.47 37–1064 (135)	147.31 ± 87.18 49.0–685.0 (111)	0.1884	0.0278	N/A	0.04 ^a^ 0.319 ^b^ 1.0 ^c^
**HbA1c** (50/213)	**On admission**	%	7.4 ± 0 7.4–7.4 (1)	7.61 ± 2.32 4.2–14.9 (126)	7.89 ± 2.61 5.6–16.6 (17)	7.44 ± 2.06 4.8–14.9 (58)	7.56 ± 1.93 5.1–13.7 (32)	6.94 ± 1.54 5.3–11.4 (29)	N/A.	0.1712	N/A.	N/A
**Urea** (217/1640)	**On admission**	mg/dL	60.58 ± 42.36 30–179 (12)	42.65 ± 35.86 5.0–307.0 (1133)	62.46 ± 48.71 17–271 (67)	63.78 ± 49.67 8.0–353.0 (388)	76.67 ± 57.07 17–369 (138)	77.92 ± 49.74 12.0–256.0 (119)	0.1519	<0.0001	N/A	<0.0001 ^a,b^ 0.033 ^c^
**Creatinine** (225/1736)	**On admission**		2.0 ± 2.84 0.51–10.84 (12)	1.14 ± 1.15 0.26–14.87 (1204)	1.93 ± 2.02 0.58–12.66 (70)	1.37 ± 1.12 0.48–9.56 (408)	1.91 ± 1.63 0.46–11.3 (143)	1.7 ± 1.53 0.44–9.49 (124)	0.9915	<0.0001	N/A	0.001 ^a^ 0.0004 ^b^ 0.074 ^c^
	**On discharge**		1.05 ± 0.41 0.55–2.06 (12)	1.08 ± 1.06 0.26–14.87 (1204)	1.97 ± 2.21 0.43–12.35 (70)	1.34 ± 1.13 0.45–9.09 (408)	1.79 ± 1.44 0.43–8.24 (143)	1.51 ± 1.4 0.43–9.27 (124)	<0.0001	<0.0001	0.006**^a^** 0.0002**^b^** 0.871 **^c^**	<0.0001 ^a^ 0.003 ^b^ 0.455 ^c^
**eGFR** (225/1731)	**On admission**	ml/min/1.73 m^2^	75.33 ± 46.57 5.0–170.0 (12)	85.11 ± 34.19 0–433.0 (1199)	60.4 ± 33.5 4.0–149.0 (70)	62.29 ± 27.91 4–137.0 (408)	52.52 ± 30.09 5.0–180.0 (143)	52.66 ± 28.92 5.0–145.0 (124)	0.1024	<0.0001	N/A	<0.0001 ^a,b^ 0.004 ^c^
	**On discharge**		86.5 ± 36.14 26.0–156.0 (12)	89.37 ± 34.73 0–433.0 (1199)	65.84 ± 38.38 4.0–208.0 (70)	65.37 ± 29.56 4.0–172.0 (408)	56.97 ± 34.53 5.0–209.0 (143)	60.07 ± 31.49 5.0–183.0 (124)	0.0209	<0.0001	0.198**^a^** 0.043**^b^** 0.234**^c^**	<0.0001 ^a,b^ 0.222 ^c^
**Total protein** (96/510)	**On admission**	g/L	5.17 ± 1.23 3.8–6.3 (3)	6.07 ± 0.84 3.5–8.2 (328)	6.25 ± 0.96 4.3–9.5 (24)	5.88 ± 0.9 3.6–8.7 (128)	5.84 ± 0.8 3.6–8.2 (69)	5.61 ± 0.95 3.3–8.1 (54)	0.2224	0.0017	N/A	0.098 ^a^ 0.004 ^b^ 0.186 ^c^
**Albumin** (110/553)	**On admission**	g/L	3.0 ± 0.2 2.8–3.2 (3)	3.17 ± 0.6 1.5–5.1 (371)	3.23 ± 0.52 2.1–4.4 (32)	3.07 ± 0.56 1.1–4.3 (128)	3.08 ± 0.57 1.7–4.9 (75)	2.82 ± 0.63 0.7–3.9 (54)	0.3433	0.0006	N/A	0.231 ^a^ 0.0008 ^b^ 0.032 ^c^
**AST** (178/1263)	**On admission**	IU/L	46.29 ± 40.36 12–124 (7)	60.24 ± 114.4 5.0–2405.0 (876)	61.11 ± 56.3 7–260 (57)	67.56 ± 281.4 8.0–4776.0 (290)	84.99 ± 365.37 10–3866 (114)	88.15 ± 271.86 8.0–2518.0 (97)	0.5289	0.5613	N/A	N/A
**ALT** (193/1395)	**On admission**	IU/L	62.1 ± 64.15 15–236 (10)	54.85 ± 93.11 4.0–1411.0 (962)	44.71 ± 38.95 4–193 (63)	49.87 ± 206.79 5.0–3700.0 (328)	45.28 ± 133.71 8–1361 (120)	58.43 ± 154.44 5.0–1315.0 (105)	0.7162	0.8697	N/A	N/A
**LDH** (141/1090)	**On admission**	U/L	345.67 ± 169.25 123.0–575.0 (6)	430.64 ± 379.34 50.0–7100.0 (770)	445.65 ± 255.92 119–1172.0 (46)	380.85 ± 188.63 44.0–1357.0 (240)	400.87 ± 258.01 71.0–1863.0 (89)	487.93 ± 1035.15 106.0–9505.0 (80)	0.4289	0.0208	N/A	0.018 ^a^ 0.876 ^b^ 0.629 ^c^
**Cardiac biomarkers**												
**NT-proBNP** (80/299)	**On admission**	ng/mL	917.1 ± 0 917.1–917.1 (1)	1894.45± 7801.53 12.0–70,000.0 (171)	12,180.61 ± 20,072.57 290–70,000 (15)	7893.21 ± 13,570.58 18.2–70,000.0 (94)	15,093.74 ± 20,598.65 391.3–70,000 (64)	12,292.06 ± 15,896.88 119.6–68,915.1 (34)	N/A	<0.0001	N/A	0.0004 ^a^ 0.002 ^b^ 0.331 ^c^
**Troponin I** (159/1015)	**On admission**		180.8 ± 219.75 8.1–542.5 (8)	136.11 ± 812.42 0–11,758.2 (670)	4399.36 ± 20,128.72 2.4–125,592.6 (44)	1252.73 ± 9482.42 1.0–109,359.5 (261)	898.68 ± 2864.92 3.9–21,022.9 (107)	587.21 ± 2311.75 3.3–18,309.3 (84)	0.0215	0.0376	0.355**^a^** 0.037**^b^** 0.49**^c^**	0.141 ^a^ 0.184 ^b^ 0.551 ^c^
		≤3-fold upper range K 46.8 M 102.6	5/8 (62.5%)	560/670 (83.58%)	33/44 (75.0%)	176/261 (67.43%)	65/107 (60.75%)	46/84 (54.76%)	0.2519	<0.0001	N/A	<0.0001 ^a,b^ 0.1438**^c^**
		>3-fold upper range K 46.8 M 102.6	3/8 (37.5%)	110/670 (16.42%)	11/44 (25.0%)	85/261 (32.57%)	42/107 (39.25%)	38/84 (45.24%)				
	**On discharge**		184.61 ± 227.67 8.1–542.5 (8)	116.05 ± 831.78 0.2–12,391.6 (670)	1662.07 ± 6227.21 2.6–36,541.1 (44)	1898.37 ± 14,149.34 0.8–174,652.6 (261)	825.42 ± 3224.71 3.9–29,828.3 (107)	455.98 ± 2095.43 1.8–18,309.3 (84)	0.0493	0.0465	0.27 ^a^ 0.119 ^b^ 0.676 ^c^	0.106 ^a^ 0.309 ^b^ 0.25 ^c^
**LDL-cholesterol** (76/373)		mg/dL	70.33 ± 30.09 51–105 (3)	100.46 ± 51.02 6.0–510.0 (229)	73.12 ± 33.82 17–143 (26)	90.7 ± 41.52 23.0–230.0 (103)	69.57 ± 45.91 6.0–210.0 (47)	80.88 ± 37.36 14.0–187.0 (41)	0.9378	0.0113	0.52 ^a^ 0.019 ^b^ 0.219 ^c^	0.159 ^a^ 0.013 ^b^ 0.357 ^c^
**HDL-cholesterol** (73/378)		mg/dL	31.33 ± 11.02 24–44 (3)	40.09 ± 16.12 2.0–120.0 (233)	35.12 ± 11.02 16–56 (25)	41.4 ± 16.05 7.0–110.0 (104)	37.04 ± 15.52 17–79.0 (45)	37.39 ± 14.56 8.0–72.0 (41)	0.6916	0.3563	N/A	N/A
**Triglycerides** (104/535)		mg/dL	90.5 ± 0.71 90–91 (2)	179.56 ± 124.26 40.0–1100.0 (357)	153.53 ± 104.59 61–550 (34)	142.09 ± 94.45 48.0–595.0 (130)	129.6 ± 66.52 46–413 (68)	128.63 ± 54.86 51.0–282.0 (48)	<0.0001	<0.0001	0.004 ^a^ <0.0001 ^b^ 0.45 ^c^	0.001 ^a^ <0.0001 ^b^ 0.47 ^c^
**Hormones**												
**TSH** (114/706)		mIU/L	2.23 ± 3.22 0.28–5.94 (3)	1.34 ± 1.52 0–18.6 (438)	1.53 ± 1.92 0.01–10.26 (38)	1.56 ± 2.56 0.01–28.81 (194)	1.5 ± 1.63 0–11.16 (73)	2.86 ± 5.27 0–38.24 (74)	0.9333	0.0317	N/A	0.511 ^a^ 0.042 ^b^ 0.109 ^c^

Continuous variables are presented as: mean ± SD range (minimum–maximum) and number of non-missing values. Categorized variables are presented as a number with a percentage. Information about the numbers with valid values is provided in the left column. Abbreviations: CAD—coronary artery disease, OMNIBUS—analysis of variance, N—valid measurements, n—number of patients with parameter above cut-off point, SD—standard deviation, N/A—non-applicable, a—low risk vs. medium risk, b—low risk vs. high risk, c—medium risk vs. high risk.

**Table 5:** Therapies applied during hospitalization in the studied cohort.

Variables, Units (N) (CAD/non-CAD)	Low Risk (0–1)		Medium Risk (2–3)		High Risk (>4)		OMNIBUS *p* Value		*p* Value (For Post Hoc Analysis)	
	n/N (% of Risk Category)		n/N (% of Risk Category)		n/N (% of Risk Category)					
	CAD	Non- CAD	CAD	Non- CAD	CAD	Non- CAD	CAD	Non- CAD	CAD	Non- CAD
Applied Treatment and Procedures										
**Systemic corticosteroid** (228/1955)	7/12 (58.33%)	701/1405 (49.89%)	42/72 (58.33%)	204/420 (48.57%)	75/144 (52.08%)	67/130 (51.54%)	0.6586	0.8145	N/A	N/A
**Convalescent plasma** (228/1955)	2/12 (16.67%)	165/1405 (11.74%)	10/72 (13.89%)	31/420 (7.38%)	17/144 (11.81%)	14/130 (10.77%)	0.6959	**0.0404**	N/A	**0.0436**^a^ 1.0 ^b^ 0.8831 ^c^
**Tocilizumab** (228/1955)	0/12 (0%)	22/1405 (1.57%)	0/72 (0%)	2/420 (0.48%)	0/144 (0%)	1/130 (0.77%)	1.0	0.1866	N/A	N/A
**Remdesivir** (228/1955)	2/12 (16.67%)	234/1405 (16.65%)	10/72 (13.89%)	62/420 (14.76%)	22/144 (15.28%)	13/130 (10.0%)	0.9042	0.1109	N/A	N/A
**Antibiotic** (228/1955)	8/12 (66.67%)	738/1405 (52.53%)	52/72 (72.22%)	251/420 (59.76%)	107/144 (74.31%)	84/130 (64.62%)	0.765	**0.0023**	N/A	**0.0318**^a^ **0.0321**^b^ 1.0 ^c^

Categorized variables are presented as a number with a percentage. Information about the numbers with valid values is provided in the left column. Abbreviations: CAD—coronary artery disease, OMNIBUS—analysis of variance, N—valid measurements, n—number of patients with parameter above cut-off point, SD—standard deviation, N/A—non-applicable, a—low risk vs. medium risk, b—low risk vs. high risk, c—medium risk vs. high risk.

**Table 6:** Applied treatment and procedures.

	Low Risk (0–1) n/N		Medium Risk (2–3) n/N		High Risk (≥4) n/N		OMNIBUS *p* Value		*p* Value (For Post Hoc Analysis)	
	Mean ± SD Min–Max (N) or n/N (% of Risk Category)		Mean ± SD Min–Max (N) or n/N (% of Risk Category)		Mean ± SD Min–Max (N) or n/N (% of Risk Category)					
	CAD	Non- CAD	CAD	Non- CAD	CAD	Non- CAD	CAD	Non- CAD	CAD	Non- CAD
Applied Treatment and Procedures										
**The most advanced respiratory support during hospitalization: no oxygen** (228/1952) **Low-flow oxygen support** (228/1952) **High-flow nasal cannula** **non-invasive ventilation** (228/1952) **Invasive ventilation** (228/1952)	5/12 (41.67%) 2/12 (16.67%) 2/12 (16.67%) 2/12 (16.67%)	736/1403 (52.46%) 449/1403 (32.0%) 63/1403 (4.49%) 139/1403 (9.91%)	30/72 (41.67%) 23/72 (31.94%) 4/72 (5.56%) 14/72 (19.44%)	172/419 (41.05%) 165/419 (39.38%) 35/419 (8.35%) 35/419 (8.35%)	43/144 (29.86%) 59/144 (40.97%) 18/144 (12.5%) 16/144 (11.11%)	46/130 (35.38%) 65/130 (50.0%) 9/130 (6.92%) 6/130 (4.62%)	0.0799	**<0.0001**	N/A	**<0.0001**^a,b^ 0.7256 ^c^
**Oxygenation parameters during qualification for advanced respiratory support:** SpO2% (68/563)	92 ± 0 92–92 (1)	90.63 ± 7.89 50–100 (409)	86.82 ± 8.05 72–97 (17)	86.45 ± 9.97 55–99 (116)	84.97 ± 10.9 59–99 (50)	86.39 ± 8.65 65–99 (38)	N/A	**<0.0001**	N/A	**0.0002**^a^ **0.016**^b^ 0.999 ^c^
**Therapy with catecholamines** (228/1955)	1/12 (8.33%)	130/1405 (9.25%)	11/72 (15.28%)	34/420 (8.1%)	26/144 (18.06%)	16/130 (12.31%)	0.7869	0.3465	N/A	
**Coronary revascularization** **or/and an indication for coronary revascularization** (228/1955)	0/12 (0%)	10/1405 (0.71%)	3/72 (4.17%)	9/420 (2.14%)	7/144 (4.86%)	2/130 (1.54%)	1.0	**0.0343**	N/A	**0.0697**^a^ 0.8105 ^b^ 1.0 ^c^
**Hemodialysis** (228/1955)	0/12 (0%)	46/1405 (3.27%)	5/72 (6.94%)	8/420 (1.9%)	10/144 (6.94%)	2/130 (1.54%)	1.0	0.269	N/A	N/A

Continuous variables are presented as: mean ± SD, range (minimum–maximum) and number of non-missing values. Categorized variables are presented as a number with a percentage. Information about the numbers with valid values is provided in the left column. Abbreviations: CAD—coronary artery disease, OMNIBUS—analysis of variance, N—valid measurements, n—number of patients with parameter above cut-off point, SD—standard deviation, N/A—non-applicable, a—low risk vs. medium risk, b—low risk vs. high risk, c—medium risk vs. high risk.

**Table 7:** Total and in-hospital all-cause mortality in the C_2_HEST risk strata in CAD and non-CAD cohorts.

Variables, Units (N) (CAD/non-CAD)	Low Risk (0–1)		Medium Risk (2–3)		High Risk (≥4)		OMNIBUS *p* Value		*p* Value (For Post Hoc Analysis)	
	n/N (% of Risk Category)		n/N (% of Risk Category)		n/N (% of Risk Category)					
	CAD	Non- CAD	CAD	Non- CAD	CAD	Non- CAD	CAD	Non- CAD	CAD	Non- CAD
All-Cause Mortality Rate										
**In-hospital mortality** (228/1955)	4/12 (33.33%)	115/1405 (8.19%)	15/72 (20.83%)	95/420 (22.62%)	62/144 (43.06%)	35/130 (26.92%)	**0.0038**	**<0.0001**	1.0 ^a^ 1.0 ^b^ **0.0044** ^c^	**<0.0001**^a,b^ 1.0 ^c^
**3-month mortality** (228/1955)	5/12 (41.67%)	197/1405 (14.02%)	24/72 (33.33%)	174/420 (41.43%)	81/144 (56.25%)	66/130 (50.77%)	**0.0058**	**<0.0001**	1.0 ^a^ 1.0 ^b^ **0.0073** ^c^	**<0.0001**^a,b^ 0.2275 ^c^
**6-month mortality** (228/1955)	5/12 (55.56%)	209/1405 (24.36%)	26/72 (42.62%)	182/420 (56.35%)	85/144 (65.89%)	71/130 (64.55%)	**0.0085**	**<0.0001**	1.0 ^a^ 1.0 ^b^ **0.0085** ^c^	**<0.0001**^a,b^ 0.4891 ^c^

Continuous variables are presented as: mean ± SD, range (minimum–maximum) and number of non-missing values. Categorized variables are presented as a number with a percentage. Information about the numbers with valid values is provided in the left column. Abbreviations: CAD—coronary artery disease, OMNIBUS—analysis of variance, N—valid measurements, n—number of patients with parameter above cut-off point, SD—standard deviation, N/A—non-applicable, a—low risk vs. medium risk, b—low risk vs. high risk, c—medium risk vs. high risk.

**Table 8:** Total all-cause-death hazard ratios for C_2_HEST risk stratification in CAD cohort.

Total Death			
	**HR**	**95% CI**	***p* Value**
**Overall**	1.1494	1.0340–1.2776	**0.0099**
**Risk Strata**			
**Low vs. medium risk**	0.7874	0.3038–2.0407	0.6228
**Low vs. high risk**	**1.4685**	0.5961–3.6177	0.4035

**Table 9:** Total all-cause-death hazard ratios for C_2_HEST risk stratification in non-CAD cohort.

Total Death			
	**HR**	**95% CI**	***p* Value**
**Overall**	1.4764	1.4038–1.5527	**<0.0001**
**Risk Strata**			
**Low vs. medium risk**	3.5959	2.9546–4.3764	**<0.0001**
**Low vs. high risk**	**4.8465**	3.7188–6.3163	**<0.0001**

**Table 10:** Associations of individual C_2_HEST score components with mortality in CAD cohort.

	Component	HR	CI Min.	CI Max.	*p* Value
**All-cause mortality**	Coronary artery disease	N/A	N/A	N/A	N/A
	COPD	1.1924	0.6803	2.0899	0.5388
	Age > 75	1.7757	1.2366	2.5499	**0.0019**
	Thyroid disease	0.7839	0.4726	1.3003	0.3457
	Hypertension	0.8471	0.5337	1.3445	0.4814
	HFrEF	1.2207	0.8390	1.7760	0.2972

COPD—chronic obstructive pulmonary disease, HFrEF—heart failure with reduced ejection fraction.

**Table 11:** Associations of individual C_2_HEST score components with mortality in non-CAD cohort.

	Component	HR	CI Min.	CI Max.	*p* Value
**All-cause mortality**	Coronary artery disease	N/A	N/A	N/A	N/A
	COPD	1.7620	1.1896	2.6098	**0.0047**
	Age > 75	2.9460	2.4086	3.6033	**<0.0001**
	Thyroid disease	0.7333	0.5298	1.0149	0.0614
	Hypertension	1.4256	1.1666	1.7422	**0.0005**
	HFrEF	1.7073	1.3160	2.2149	**0.0001**

COPD—chronic obstructive pulmonary disease, HFrEF—heart failure with reduced ejection fraction.

**Table 12:** Log-rank statistics for matching the C_2_HEST risk strata for all-cause hospital mortality in CAD cohort.

	h2	h3	h4	h5	h6	h7	h8
m1	0.4262	2.7269	2.9888	2.2412	1.26	−0.0248	N/A
m2		7.9148	9.2053	5.0264	1.6577	0.1843	0.4262
m3			9.2833	8.8761	7.576	7.6551	2.7269
m4				**9.5121**	8.9332	9.3478	2.9888
m5					5.6817	5.9157	2.2412
m6						2.1085	1.2600
m7							−0.0248

m—medium, h—high.

**Table 13:** Log-rank statistics for matching the C_2_HEST risk strata for all causes mortality in non-CAD cohort.

	h2	h3	h4	h5	h6	h7	h8
m1	**237.344**	193.6023	170.6614	158.5977	132.5961	11.5029	11.5029
m2		226.8814	232.9099	232.4957	225.913	15.0212	15.0212
m3			151.9224	151.6698	146.3188	12.0786	12.0786
m4				79.4099	79.9497	8.8469	8.8469
m5					53.0824	7.0857	7.0857
m6						1.3654	1.3654
m7							2.769

m—medium, h—high.

**Table 14:** Clinical non-fatal events and hospitalization outcomes in the C_2_HEST risk strata in CAD and non-CAD cohorts.

Variables, Units (N) (CAD/non-CAD)	Low Risk (0–1)		Medium Risk (2–3)		High Risk (≥4)		OMNIBUS *p* Value		*p* Value (For Post Hoc Analysis)	
	Mean ± SD Min–Ma x(N) or n/N (% of Risk Category)		Mean ± SD Min–Ma x(N) or n/N (% of Risk Category)		Mean ± SD Min–Ma x(N) or n/N (% of Risk Category)					
	CAD	Non-CAD	CAD	Non-CAD	CAD	Non-CAD	CAD	Non-CAD	CAD	Non-CAD
**Hospitalization**										
**Duration of hospitalization, days** (228/1955)	6.67 ± 5.43 1–16 (12)	11.53 ± 13.7 1–131 (1405)	13.86 ± 13.32 1–69 (72)	13.06 ± 13.66 1–124 (420)	15.39 ± 15.52 1–121 (144)	16.85 ± 16.29 1–87 (130)	0.0003	0.0005	0.007 ^a^ 0.0005 ^b^ 0.733 ^c^	0.109 ^a^ 0.001 ^b^ 0.045 ^c^
**Admission to ICU** (228/1955)	1/12 (8.33%)	149/1405 (10.61%)	11/72 (15.28%)	27/420 (6.43%)	19/144 (13.19%)	8/130 (6.15%)	0.8945	0.0154	N/A	0.043 ^a^ 0.440 ^b^ 1.0 ^c^
**End of hospitalization** **death** (228/1955) **Discharge to home—full recovery** **Transfer to another hospital—worsening** **Transfer to another hospital—in recovery**	4/12 (33.33%) 3/12 (25.0%) 3/12 (25.0%) 2/12 (16.67%)	115/1405 (8.18%) 990/1405 (70.46%) 136/1405 (9.68%) 164/1405 (11.67%)	15/72 (20.83%) 40/72 (55.56%) 29/72 (12.5%) 8/72 (11.11%)	95/420 (22.62%) 180/420 (42.86%) 788/420 (20.95%) 57/420 (13.57%)	62/144 (43.06%) 45/144 (31.25%) 20/144 (13.89%) 17/144 (11.81%)	35/130 (25.92%) 58/130 (44.62%) 24/130 (18.46%) 13/130 (10.0%)	0.0076	<0.0001	0.5166 ^a^ 1.0 ^b^ 0.0082 ^c^	<0.0001 ^a,b^ 1.0 ^c^
**Aborted cardiac arrest** (228/1955)	1/12 (8.33%)	14/1405 (0.99%)	1/72 (1.39%)	2/420 (0.48%)	4/144 (2.78%)	2/130 (1.53%)	0.3288	0.4044	N/A	N/A
**Shock** (228/1955)	0/12 (0%)	108/1405 (7.69%)	9/72 (12.5%)	37/420 (8.81%)	19/144 (13.19%)	14/130 (10.77%)	0.5706	0.3984	N/A	N/A
**Hypovolemic shock** (228/1955)	0/12 (0%)	22/1405 (1.57%)	0/72 (0%)	7/420 (1.67%)	0/144 (0%)	6/130 (4.62%)	<0.0001	0.0574	<0.0001 ^a,b,c^	N/A
**Cardiogenic shock** (228/1955)	0/12 (0%)	7/1405 (0.5%)	2/72 (2.78%)	9/420 (2.14%)	9/144 (6.25%)	5/130 (3.85%)	0.5501	0.0002	N/A	0.012 ^a^ 0.0059 ^b^ 1.0 ^c^
**Septic shock** (228/1955)	0/12 (0%)	88/1405 (6.26%)	6/72 (8.33%)	24/420 (5.71%)	16/144 (11.11%)	6/130 (4.62%)	0.6393	0.7163	N/A	N/A
**Pulmonary embolism** (228/1955)	0/12 (0%)	78/1405 (5.55%)	1/72 (1.39%)	27/420 (6.43%)	8/144 (5.55%)	7/130 (5.38%)	0.6343	0.931	N/A	N/A
**Myocardial infarction** (228/1955)	0/12 (0%)	8/1405 (0.57%)	2/72 (2.78%)	8/420 (1.9%)	5/144 (3.47%)	3/130 (2.31%)	1.0	0.0096	N/A	0.0487 ^a^ 0.1775 ^b^ 1.0 ^c^
**Myocardial injury** (159/1015)	3/8 (37.5%)	110/670 (16.42%)	13/44 (29.55%)	85/261 (32.57%)	49/107 (45.79%)	38/84 (45.24%)	0.1696	<0.0001	N/A	<0.0001 ^a,b^ 0.1438 ^c^
**Acute heart failure** (228/1955)	1/12 (8.33%)	7/1405 (0.5%)	0/72 (0%)	22/420 (5.24%)	27/144 (18.75%)	19/130 (14.62%)	<0.0001	<0.0001	0.4286 ^a^ 1.0 ^b^ <0.0001 ^c^	<0.0001 ^a,b^ 0.0027 ^c^
**Stroke/TIA** (228/1955)	0/12 (0%)	18/1405 (1.28%)	3/72 (4.17%)	16/420 (3.81%)	4/144 (2.78%)	3/130 (2.31%)	0.7877	0.004	N/A	0.0052 ^a^ 1.0 ^b,c^
**New cognitive disorders** (228/1955)	0/12 (0%)	38/1405 (2.7%)	3/72 (4.17%)	48/420 (11.43%)	17/144 (11.81%)	15/130 (11.54%)	0.1059	<0.0001	N/A	<0.0001 ^a,b^ 1.0 ^c^
**Pneumonia** (228/1955)	5/12 (41.67%)	670/1405 (48.19%)	39/72 (54.17%)	266/420 (63.33%)	99/144 (68.75%)	87/130 (66.92%)	0.0358	<0.0001	1.0 ^a^ 0.3187 ^b^ 0.1 ^c^	<0.0001 ^a^ 0.0002 ^b^ 1.0 ^c^
**Complete respiratory failure** (56/220)	1/3 (33.33%)	56/118 (47.46%)	7/14 (50.0%)	39/74 (52.7%)	30/39 (76.92%)	13/28 (46.43%)	0.0824	0.7439	N/A	N/A
**SIRS** (228/1886)	2/12 (16.67%)	140/1340 (10.45%)	4/72 (5.56%)	38/417 (9.11%)	19/144 (13.19%)	17/129 (13.18%)	0.1331	0.4035	N/A	N/A
**Sepsis** (91/793)	0/2 (0%)	9/574 (1.57%)	1/28 (3.57%)	6/155 (3.87%)	2/61 (3.28%)	5/64 (7.81%)	1.0	0.0077	N/A	0.3098 ^a^ 0.0256 ^b^ 0.9164 ^c^
**Acute kidney injury** (228/1955)	0/12 (0%)	110/1405 (7.83%)	13/72 (18.06%)	54/420 (12.86%)	38/144 (26.39%)	21/130 (16.15%)	0.053	0.0002	N/A	0.0065 ^a^ 0.0061 ^b^ 1.0 ^c^
**Acute liver dysfunction** (216/1758)	0/12 (0%)	30/1244 (2.41%)	2/68 (2.94%)	20/397 (5.04%)	9/136 (6.62%)	5/117 (4.27%)	0.4767	0.0221	N/A	0.03423 ^a^ 0.6518 ^b^ 1.0 ^c^
**MODS** (228/1955)	0/12 (0%)	21/1405 (1.49%)	1/72 (1.39%)	7/420 (1.67%)	5/144 (3.47%)	3/130 (2.31%)	0.7595	0.6267	N/A	N/A
**Lactic acidosis** (53/192)	1/3 (33.33%)	8/102 (7.85%)	0/13 (0%)	5/66 (7.58%)	6/37 (16.22%)	2/24 (8.33%)	0.1835	1.0	N/A	N/A
**Bleedings** (228/1955)	0/12 (0%)	64/1405 (4.56%)	5/72 (6.94%)	20/420 (4.76%	12/144 (8.33%)	13/130 (10.0%)	0.8366	0.0232	N/A	1.0 ^a^ 0.0361 ^b^ 0.141 ^c^

Continuous variables are presented as: mean ± SD, range (minimum–maximum) and number of non-missing values. Categorized variables are presented as a number with a percentage. Information about the numbers with valid values is provided in the left column. Abbreviations: CAD—coronary artery disease, OMNIBUS—analysis of variance, N—valid measurements, n—number of patients with parameter above cut-off point, SD—standard deviation, ICU—intensive care unit, TIA—transient ischemic attack, SIRS—systemic inflammatory response syndrome, MODS—multiple organ dysfunction syndrome, N/A—non-applicable, a—low risk vs. medium risk, b—low risk vs. high risk, c—medium risk vs. high risk.

## Data Availability

The datasets used and/or analyzed during the current study are available from the corresponding author upon reasonable request.

## Supplementary Materials

The following supporting information can be downloaded at: https://www.mdpi.com/article/10.3390/v14081771/s1, Table S1: Influence of age in the CAD and non-CAD groups on the all-cause mortality; Figure S1: Predictive value of C_2_HEST components on the endpoints in the CAD and non-CAD groups.
