# Supplementary material for: The Usefulness of the C2HEST Risk Score in Predicting Clinical Outcomes among Hospitalized Subjects with COVID-19 and Coronary Artery Disease
Source: Viruses. 2022 Aug 14;14(8):1771. doi: 10.3390/v14081771 (PMC9415686; doi:10.3390/v14081771)
Supplement: Supplementary file 1 [file viruses-14-01771-s001.zip › viruses-1854651-supplementary.pdf]

As in the previous analysis, it has been shown that age was among the most important and significant component of the C2HEST score for the survival analysis (in the *Cox* model), a bifactorial *Cox* model has been built. The analysis of the influence of age in the CAD and non-CAD groups on the all-cause mortality is presented in the Tables below:

Supplementary Table S1. Influence of age in the CAD and non-CAD groups on the all-cause mortality

A. CAD group

	Hazard ratio	Lower CI limit	Upper CI limit	p-value
C2HEST (points)	1.006	0.8826	1.146	0.9304
Age (years)	1.041	1.0160	1.066	**0.0012**

B. Non-CAD group

**Non-CAD group**	Hazard ratio	Lower CI limit	Upper CI limit	p-value
C2HEST (points)	1.027	0.9503	1.110	0.499
Age (years)	1.053	1.0442	1.062	**<0.001**

Age was the strongest predictor of survival/mortality in both cohorts. Noteworthy, since the multivariate models do not analyze the relationship, but the predictive value, the non-significant p-value for C2HEST does not indicate that the survival is independent on the C2HEST components as well as on C2HEST score value – the univariate model has confirmed the presence of such relationship. The C2HEST score has still better predictive value for the endpoints than its components solely, as presented for both cohorts in the Figures below:

Supplementary Figure S1. Predictive value of C2HEST components on the endpoints in the CAD and non-CAD groups.

A. CAD group

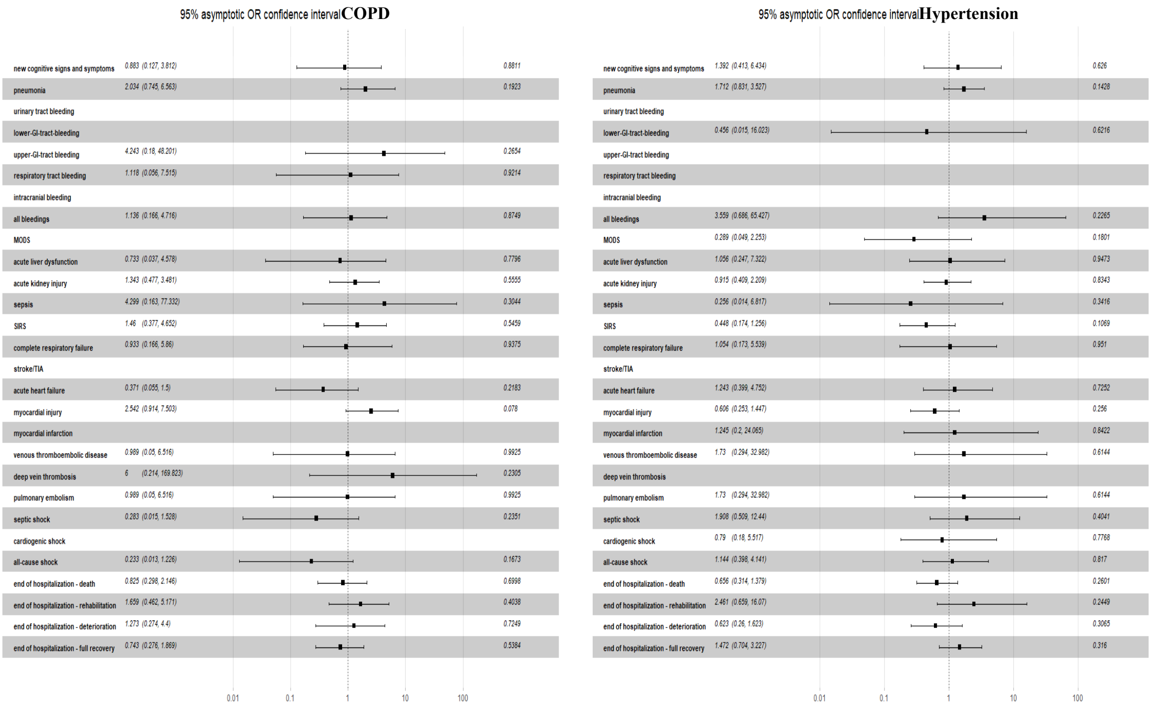

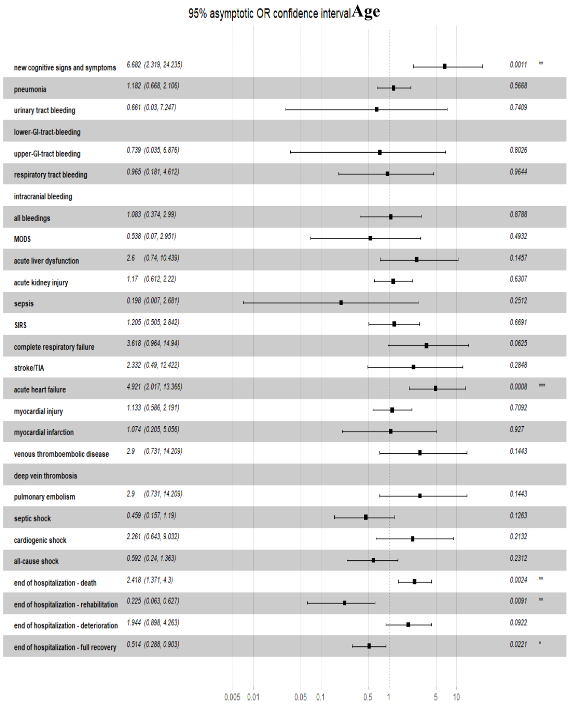

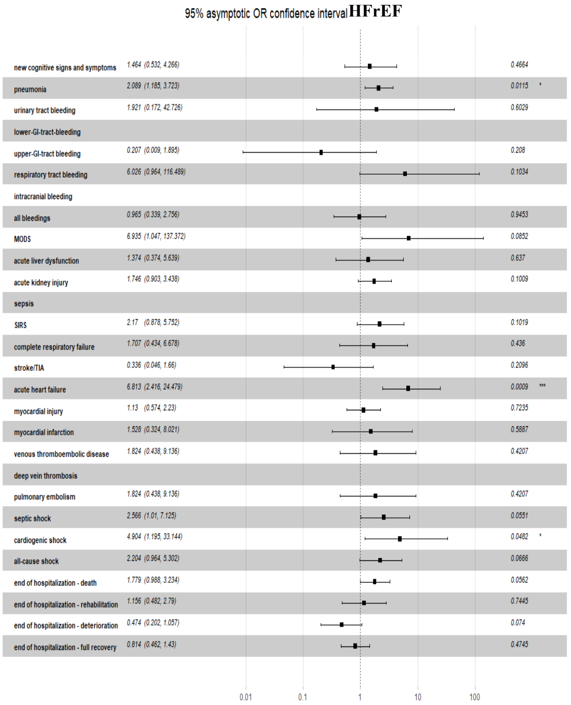

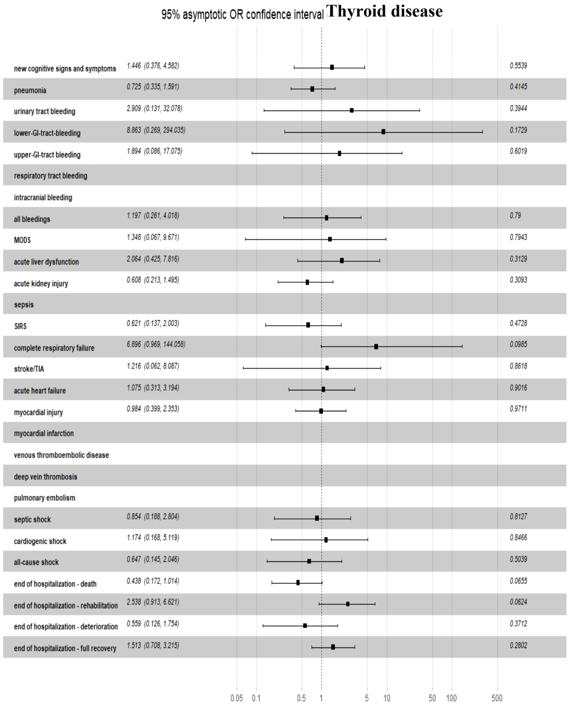

p-value	*Significance code*
<0.0001	******
<0.001	*****
<0.01	****
<0.05	***
>=0.05	

B. Non-CAD group:

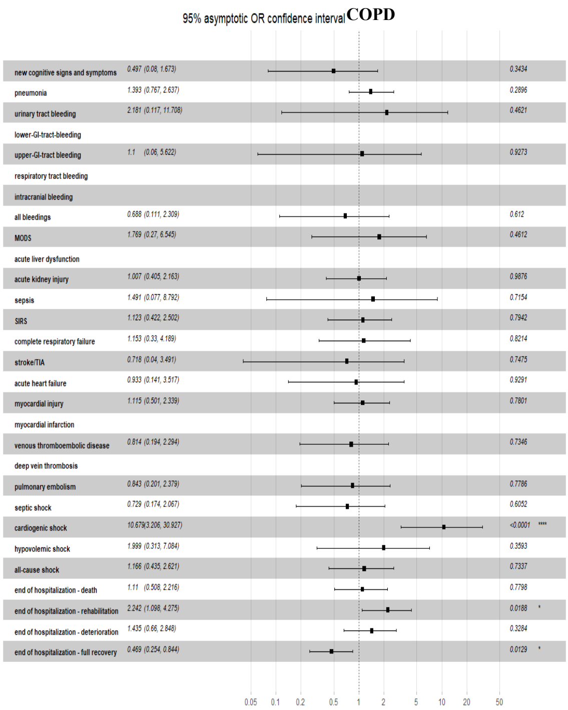
95% asymptotic OR confidence interval
COPD
new cognitive signs and symptoms 0.497 (0.08, 1.673) 0.3434
pneumonia 1.393 (0.767, 2.637) 0.2896
urinary tract bleeding 2.181 (0.117, 11.708) 0.4621
lower-GI-tract-bleeding
upper-GI-tract bleeding 1.1 (0.06, 5.622) 0.9273
respiratory tract bleeding
intracranial bleeding
all bleedings 0.688 (0.111, 2.309) 0.612
MODS 1.769 (0.27, 6.545) 0.4612
acute liver dysfunction
acute kidney injury 1.007 (0.405, 2.163) 0.9876
sepsis 1.491 (0.077, 8.792) 0.7154
SIRS 1.123 (0.422, 2.502) 0.7942
complete respiratory failure 1.153 (0.33, 4.189) 0.8214
stroke/TIA 0.718 (0.04, 3.491) 0.7475
acute heart failure 0.933 (0.141, 3.517) 0.9291
myocardial injury 1.115 (0.501, 2.339) 0.7801
myocardial infarction
venous thromboembolic disease 0.814 (0.194, 2.294) 0.7346
deep vein thrombosis
pulmonary embolism 0.843 (0.201, 2.379) 0.7786
septic shock 0.729 (0.174, 2.067) 0.6052
cardiogenic shock 10.679(3.206, 30.927) <0.0001 ****
hypovolemic shock 1.999 (0.313, 7.084) 0.3593
all-cause shock 1.166 (0.435, 2.621) 0.7337
end of hospitalization - death 1.11 (0.508, 2.216) 0.7798
end of hospitalization - rehabilitation 2.242 (1.098, 4.275) 0.0188 *
end of hospitalization - deterioration 1.435 (0.66, 2.848) 0.3284
end of hospitalization - full recovery 0.469 (0.254, 0.844) 0.0129 *
0.05 0.1 0.2 0.5 1 2 5 10 20 50

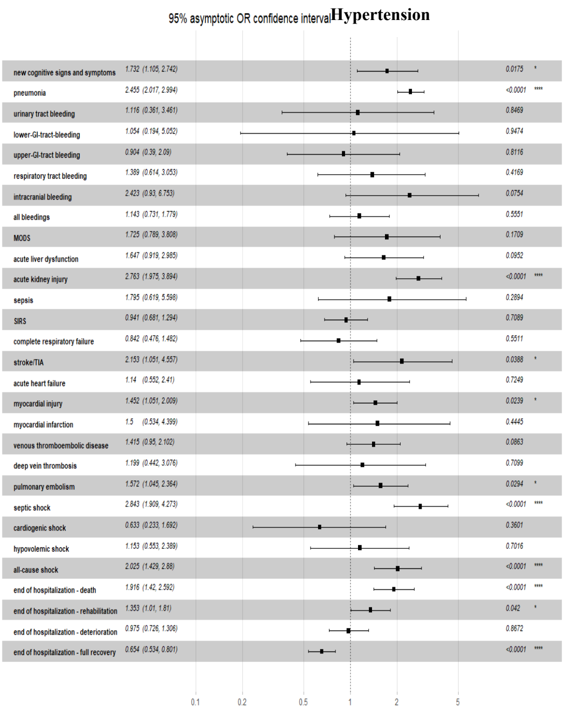
95% asymptotic OR confidence interval
Hypertension
new cognitive signs and symptoms 1.732 (1.105, 2.742) 0.0175 *
pneumonia 2.455 (2.017, 2.994) <0.0001 ****
urinary tract bleeding 1.116 (0.361, 3.461) 0.8469
lower-GI-tract-bleeding 1.054 (0.194, 5.052) 0.9474
upper-GI-tract bleeding 0.904 (0.39, 2.09) 0.8116
respiratory tract bleeding 1.389 (0.614, 3.053) 0.4169
intracranial bleeding 2.423 (0.93, 6.753) 0.0754
all bleedings 1.143 (0.731, 1.779) 0.5551
MODS 1.725 (0.789, 3.808) 0.1709
acute liver dysfunction 1.647 (0.919, 2.985) 0.0952
acute kidney injury 2.763 (1.975, 3.894) <0.0001 ****
sepsis 1.795 (0.619, 5.598) 0.2894
SIRS 0.941 (0.681, 1.294) 0.7089
complete respiratory failure 0.842 (0.476, 1.482) 0.5511
stroke/TIA 2.153 (1.051, 4.557) 0.0388 *
acute heart failure 1.14 (0.552, 2.41) 0.7249
myocardial injury 1.452 (1.051, 2.009) 0.0239 *
myocardial infarction 1.5 (0.534, 4.399) 0.4445
venous thromboembolic disease 1.415 (0.95, 2.102) 0.0863
deep vein thrombosis 1.199 (0.442, 3.076) 0.7099
pulmonary embolism 1.572 (1.045, 2.364) 0.0294 *
septic shock 2.843 (1.909, 4.273) <0.0001 ****
cardiogenic shock 0.633 (0.233, 1.692) 0.3601
hypovolemic shock 1.153 (0.553, 2.389) 0.7016
all-cause shock 2.025 (1.429, 2.88) <0.0001 ****
end of hospitalization - death 1.916 (1.42, 2.592) <0.0001 ****
end of hospitalization - rehabilitation 1.353 (1.01, 1.81) 0.042 *
end of hospitalization - deterioration 0.975 (0.726, 1.306) 0.8672
end of hospitalization - full recovery 0.654 (0.534, 0.801) <0.0001 ****
0.1 0.2 0.5 1 2 5

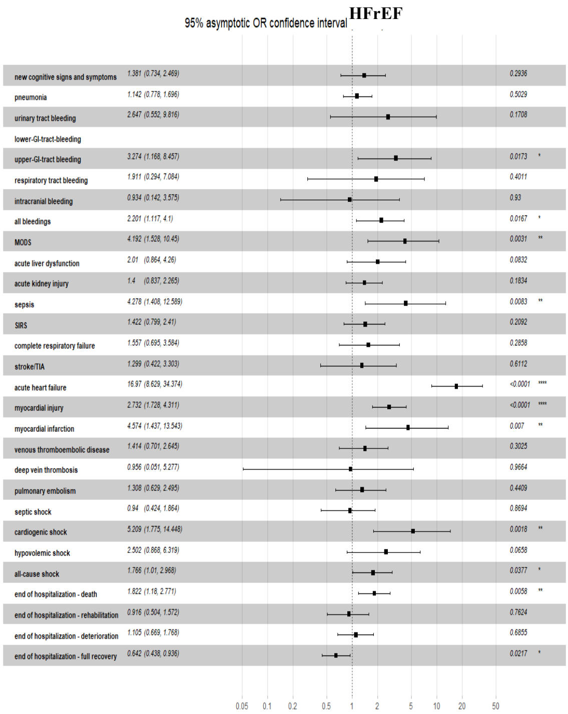
95% asymptotic OR confidence interval
HFrEF
new cognitive signs and symptoms 1.381 (0.734, 2.469) 0.2936
pneumonia 1.142 (0.778, 1.696) 0.5029
urinary tract bleeding 2.647 (0.552, 9.816) 0.1708
lower-GI-tract-bleeding
upper-GI-tract bleeding 3.274 (1.168, 8.457) 0.0173 *
respiratory tract bleeding 1.911 (0.294, 7.084) 0.4011
intracranial bleeding 0.934 (0.142, 3.575) 0.93
all bleedings 2.201 (1.117, 4.1) 0.0167 *
MODS 4.192 (1.528, 10.45) 0.0031 **
acute liver dysfunction 2.01 (0.864, 4.26) 0.0832
acute kidney injury 1.4 (0.837, 2.265) 0.1834
sepsis 4.278 (1.408, 12.589) 0.0083 **
SIRS 1.422 (0.799, 2.41) 0.2092
complete respiratory failure 1.557 (0.695, 3.584) 0.2858
stroke/TIA 1.299 (0.422, 3.303) 0.6112
acute heart failure 16.97 (8.629, 34.374) <0.0001 ****
myocardial injury 2.732 (1.728, 4.311) <0.0001 ****
myocardial infarction 4.574 (1.437, 13.543) 0.007 **
venous thromboembolic disease 1.414 (0.701, 2.645) 0.3025
deep vein thrombosis 0.956 (0.051, 5.277) 0.9664
pulmonary embolism 1.308 (0.629, 2.495) 0.4409
septic shock 0.94 (0.424, 1.864) 0.8694
cardiogenic shock 5.209 (1.775, 14.448) 0.0018 **
hypovolemic shock 2.502 (0.868, 6.319) 0.0658
all-cause shock 1.766 (1.01, 2.968) 0.0377 *
end of hospitalization - death 1.822 (1.18, 2.771) 0.0058 **
end of hospitalization - rehabilitation 0.916 (0.504, 1.572) 0.7624
end of hospitalization - deterioration 1.105 (0.669, 1.768) 0.6855
end of hospitalization - full recovery 0.642 (0.438, 0.936) 0.0217 *
0.05 0.1 0.2 0.5 1 2 5 10 20 50

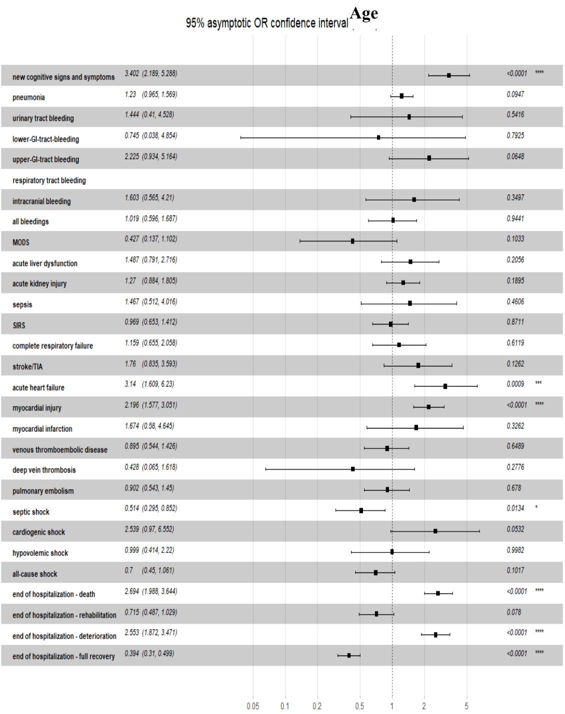
95% asymptotic OR confidence interval
Age
new cognitive signs and symptoms 3.402 (2.189, 5.288) <0.0001 ****
pneumonia 1.23 (0.965, 1.569) 0.0947
urinary tract bleeding 1.444 (0.41, 4.528) 0.5416
lower-GI-tract-bleeding 0.745 (0.038, 4.854) 0.7925
upper-GI-tract bleeding 2.225 (0.934, 5.164) 0.0648
respiratory tract bleeding
intracranial bleeding 1.603 (0.565, 4.21) 0.3497
all bleedings 1.019 (0.596, 1.687) 0.9441
MODS 0.427 (0.137, 1.102) 0.1033
acute liver dysfunction 1.487 (0.791, 2.716) 0.2056
acute kidney injury 1.27 (0.884, 1.805) 0.1895
sepsis 1.467 (0.512, 4.016) 0.4606
SIRS 0.969 (0.653, 1.412) 0.8711
complete respiratory failure 1.159 (0.655, 2.058) 0.6119
stroke/TIA 1.76 (0.835, 3.593) 0.1262
acute heart failure 3.14 (1.609, 6.23) 0.0009 ***
myocardial injury 2.196 (1.577, 3.051) <0.0001 ****
myocardial infarction 1.674 (0.58, 4.645) 0.3262
venous thromboembolic disease 0.895 (0.544, 1.426) 0.6489
deep vein thrombosis 0.428 (0.065, 1.618) 0.2776
pulmonary embolism 0.902 (0.543, 1.45) 0.678
septic shock 0.514 (0.295, 0.852) 0.0134 *
cardiogenic shock 2.539 (0.97, 6.552) 0.0532
hypovolemic shock 0.999 (0.414, 2.22) 0.9982
all-cause shock 0.7 (0.45, 1.061) 0.1017
end of hospitalization - death 2.694 (1.988, 3.644) <0.0001 ****
end of hospitalization - rehabilitation 0.715 (0.487, 1.029) 0.078
end of hospitalization - deterioration 2.553 (1.872, 3.471) <0.0001 ****
end of hospitalization - full recovery 0.394 (0.31, 0.499) <0.0001 ****
0.05 0.1 0.2 0.5 1 2 5

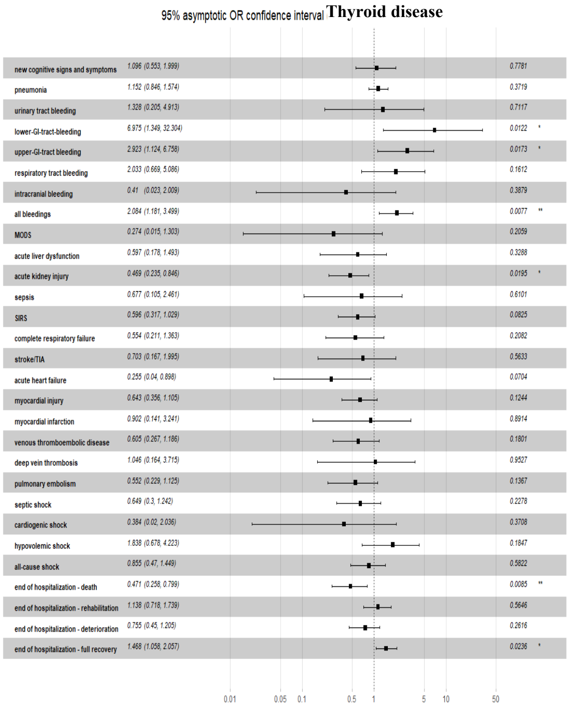

p-value	*Significance code*
<0.0001	****
<0.001	***
<0.01	**
<0.05	*
>=0.05	
